# Identification of novel genes involved in DNA damage response by screening a genome-wide *Schizosaccharomyces pombe* deletion library

**DOI:** 10.1186/1471-2164-13-662

**Published:** 2012-11-23

**Authors:** Xian Pan, Bingkun Lei, Nan Zhou, Biwei Feng, Wei Yao, Xin Zhao, Yao Yu, Hong Lu

**Affiliations:** 1 State Key Laboratory of Genetic Engineering, School of Life Sciences, and Institutes of Biomedical Sciences, Fudan University, Shanghai, 200433, China; 2 Department of Animal Science, McGill University, Ste. Anne de Bellevue, Quebec, H9X 3V9, Canada

## Abstract

**Background:**

DNA damage response (DDR) plays pivotal roles in maintaining genome integrity and stability. An effective DDR requires the involvement of hundreds of genes that compose a complicated network. Because DDR is highly conserved in evolution, studies in lower eukaryotes can provide valuable information to elucidate the mechanism in higher organisms. Fission yeast (*Schizosaccharomyces pombe*) has emerged as an excellent model for DDR research in recent years. To identify novel genes involved in DDR, we screened a genome-wide *S*. *pombe* haploid deletion library against six different DNA damage reagents. The library covered 90.5% of the nonessential genes of *S. pombe*.

**Results:**

We have identified 52 genes that were actively involved in DDR. Among the 52 genes, 20 genes were linked to DDR for the first time. Flow cytometry analysis of the repair defective mutants revealed that most of them exhibited a defect in cell cycle progression, and some caused genome instability. Microarray analysis and genetic complementation assays were carried out to characterize 6 of the novel DDR genes in more detail. Data suggested that *SPBC2A9.02* and *SPAC27D7.08c* were required for efficient DNA replication initiation because they interacted genetically with DNA replication initiation proteins Abp1 and Abp2. In addition, deletion of *sgf73*^+^, *meu29*^+^, *sec65*^+^ or *pab1*^+^ caused improper cytokinesis and DNA re-replication, which contributed to the diploidization in the mutants.

**Conclusions:**

A genome-wide screen of genes involved in DDR emphasized the key role of cell cycle control in the DDR network. Characterization of novel genes identified in the screen helps to elucidate the mechanism of the DDR network and provides valuable clues for understanding genome stability in higher eukaryotes.

## Background

Genomes are under constant threat of damage from exogenous factors and endogenous processes that result in DNA lesions. Correspondingly, cells have evolved elaborate DNA damage response (DDR) mechanisms to maintain genome integrity and stability [1]. DDR integrates the DNA-repair process with the cell cycle regulation, chromatin dynamics and programmed cell death, requiring delicate coordination of hundreds of genes [2]. Because DNA damage underlies the onset of cancer, aging, immune deficiencies, and other degenerative diseases, urgent needs of public health have made DDR a major target of study for decades [3].

DDR is highly conserved during evolution. Essential components of the DDR network, including ATM/ATR pathway, non-homologous ends joining (NHEJ) and homologous recombination (HR) repair, share homologues among almost all the eukaryotes [4]. Therefore, studies of the DDR in lower eukaryotes can provide valuable information to elucidate the mechanism in higher organisms. Because of their experimental amenabilities, budding yeast (*Saccharomyces cerevisiae*) and fission yeast (*Schizosaccharomyces pombe*) have become excellent models for DDR research [5,6]. Fission yeast separated from budding yeast about 1,000 million years ago during evolution. *S. pombe* contains about 150 metazoan-homologous genes which can’t be found in *S. cerevisiae*, and a similar number is seen when this comparison is made for *S. cerevisiae*. This emphasizes the advantage of using both yeasts for basic studies [7]. With the completion of the *Saccharomyces* Genome Deletion Project in 1999, genome-wide screens using a deletion library have become an effective way to identify novel genes involved in DDR [8]. Using such systematic screens, scientists have discovered 40 genes required for repairing DNA lesions caused by MMS [9], 31 genes involved in DDR to UV [10], and 107 new loci that influence sensitivity to γ radiation [11]. A haploid deletion library of *S. pombe* was created by Korea Research Institute of Biotechnology and Bioscience and supplied by Bioneer Corporation (http://pombe.bioneer.co.kr/). This commercial library facilitates the genome-wide screen in fission yeast. By using this library, colleagues identified 229 genes relevant to DDR, among which 23 genes were previously uncharacterized [12]. Following, an upgraded library was applied to investigate the global fitness of deletions after different kinds of DNA damage by barcode sequencing [13]. Both studies made impressive progress to gain a better understanding of DDR. However, the deletion libraries applied in these studies only covered around 70% of non-essential *S. pombe* genes. In this sense, screening a deletion library with a higher coverage of genes seemed worthwhile in order to build a more comprehensive DDR network.

In this study, we screened a *S. pombe* haploid deletion library, containing 3,235 deletions, against six different DNA damage reagents. The library represented approximately 90.5% of non-essential genes in the genome. 52 genes were identified to be closely related with DDR, 20 of which were reported for the first time. We characterized six novel DDR genes by flow cytometry and microarray analysis. Data suggest these genes might function in DNA replication and cytokinesis, providing a basis for further characterization of their roles in DDR.

## Results

### Genome-wide screen of DNA damage sensitive mutants

Six chemical reagents that can cause different kinds of DNA damage were chosen for the screen. Hydroxyurea (HU) inhibits ribonucleotide reductase, depletes nucleotides pool and thus leads to an S-phase arrest [14]. Bleomycin (BLM), a mimetic of gamma irradiation, causes double-strand breaks [15]. Methyl methanesulfonate (MMS), an alkylating agent, primarily methylates DNA on N^7^-deoxyguanine and N^3^-deoxyadenine, leading to DNA synthesis defects [9]. Camptothecin (CPT) locks topoisomerase I covalently onto the DNA and thus causes strand breaks during S phase [16]. Ultraviolent radiation (UV) results in an abnormal covalent bond between adjacent pyrimidine bases [17]. Thiabendazole (TBZ) depolymerizes the microtubule and was used to check the integrity of the spindle checkpoint [18]. Before the screen was performed, the growth of WT cells with different concentrations of DNA damaging agents were monitored. The highest concentration that did not affect the growth of WT cells was chosen for large scale screen. By using this concentration, it was easier to compare the growth with WT cells and to pick the sensitive mutants.

The screen was carried out in three rounds. First, 3,235 deletions were exposed to each DNA damage reagent in 96-well microtiter plates. 630 mutants showing sensitivities to at least one reagent were picked to create a sub-library. In the second round, mutants from the sub-library were grown in test-tubes to repeat the sensitivity assays, and 322 sensitive deletions were obtained (Additional file 1: Table S1). In the last round of the screen, 322 deletions were subjected to spot assays to quantify the sensitivities. We found that deletion of 52 genes caused viability to decrease by 25 fold or more upon treatment of at least one reagent, suggesting those genes play important roles in DDR (Table 1 and Additional file 1: Figure S1).

**Table 1:** List of genes whose deletions exhibited strong sensitivities to DNA damage reagents

**Systematic ID**	**Gene name**	**Description of the gene products**	**DNA damaging agents**	**Flow cytometry phenotype**^**a**^	**Reference**^**b**^
SPAC17A5.07c	*ulp2*^+^	SUMO deconjugating cysteine peptidase	HU, BLM, MMS, TBZ, UV	1C	[19]
SPAC1952.07	*rad1*^+^	checkpoint clamp complex protein	HU, BLM, MMS, CPT, UV	1C	[12,13,20]
SPAC23C11.15	*pst2*^+^	Clr6 histone deacetylase complex subunit	HU, BLM, MMS, TBZ, UV	2C	[21]
SPAC3C7.03c	*rhp55*^+^	RecA family ATPase	HU, BLM, MMS, TBZ, UV	2C	[12,22]
SPBC1D7.04	*mlo3*^+^	RNA annealing factor	HU, BLM, MMS, CPT, UV	W4C	[13]
SPAC1952.05	*gcn5*^+^	SAGA complex histone acetyltransferase catalytic subunit	HU, MMS, CPT, TBZ	2C	[12,13,23]
SPAC227.07c	*pab1*^+^	protein phosphatase regulatory subunit	HU, MMS, TBZ, UV	S4C	[12,13,24]
SPAC4D7.10c	*spt20*^+^	histone acetyltransferase SAGA complex subunit	HU, CPT, TBZ, UV	S4C	[23]
SPAC6G9.10c	*sen1*^+^	ATP-dependent 5' to 3' DNA/RNA helicase	HU, BLM, TBZ, UV	2C	[25]
SPBC146.13c	*myo1*^+^	myosin type I	HU, BLM, TBZ, UV	2C	[12,26]
SPBC2F12.11c	*rep2*^+^	transcriptional activator	HU, BLM, MMS, UV	1C	[13,27]
SPBC3D6.04c	*mad1*^+^	mitotic spindle checkpoint protein	HU, BLM, TBZ, UV	NC^c^	[13,28]
SPBC342.05	*crb2*^+^	DNA repair protein RAD9 homolog	HU, BLM, MMS, UV	NC	[12,13,29]
SPBC409.15		rRNA processing protein	HU, MMS, TBZ, UV	S4C	[13]
SPCC1393.05	*ers1*^+^	RNA-silencing factor	HU, BLM, TBZ, UV	2C	[13]
SPCC306.04c	*set1*^+^	histone lysine methyltransferase	HU, BLM, MMS, TBZ	2C	[13,30]
SPCC417.02	*dad5*^+^	DASH complex subunit	HU, BLM, TBZ, UV	NC	[12,13,31]
SPAC16E8.09	*scd1*^+^	Rho guanine nucleotide exchange factor	HU, BLM, TBZ	2C	[32]
SPAC4H3.05	*srs2*^+^	ATP-dependent DNA helicase	BLM, MMS, UV	1C	[12,33]
SPAC6F6.01	*cch1*^+^	calcium channel	HU, MMS, UV	2C	[12,13]
SPAC664.07c	*rad9*^+^	checkpoint clamp complex protein	HU, BLM, MMS	NC	[12,13,20]
SPBC11B10.10c	*pht1*^+^	histone H2A variant	BLM, MMS, UV	NC	[34]
SPBC13G1.08c	*ash2*^+^	Ash2-trithorax family protein, Set1 complex component	HU, BLM, TBZ	2C	[12,30]
SPBC428.08c	*clr4*^+^	histone H3 methyltransferase	HU, MMS ,TBZ	1C	[12,13,35]
SPBC660.11	*tcg1*^+^	single-stranded telomeric binding protein	HU, BLM, TBZ	2C	[13]
SPBC800.05c	*atb2*^+^	tubulin alpha 2	HU, BLM, TBZ	W4C	[13,36]
SPCC126.04c	*sgf73*^+^	histone acetyltransferase SAGA complex subunit	HU, MMS, TBZ	W4C	[12,23]
SPCC126.15c	*sec65*^+^	signal recognition particle subunit	BLM, TBZ, UV	S4C	[12,13]
SPCC162.12	*tco89*^+^	TORC1 subunit	HU, BLM, UV	NC	[37]
SPAC2F7.08c	*snf5*^+^	chromatin remodeling complex subunit	HU, MMS	1C	[38]
SPAC664.01c	*swi6*^+^	chromodomain protein	HU, TBZ	NC	[12,13,35]
SPAC19A8.11c		recombination protein	BLM	NC	[13]
SPAC3F10.02c	*trk1*^+^	potassium ion transporter	HU, BLM, CPT, TBZ, UV	2C	This study
SPAC1486.04c	*alm1*^+^	medial ring protein	HU, BLM, MMS, UV	NC	This study
SPAC17G6.06^d^	*rps2401*^+^	40S ribosomal protein	HU, BLM, MMS, UV	2C	This study
SPBC2A9.02^e^		NAD dependent epimerase/dehydratase family protein	HU, BLM, MMS, UV	1C	This study
SPCC63.02c	*aah3*^+^	alpha-amylase homolog	HU, BLM, TBZ, UV	W4C	This study
SPAC14C4.05c^d^	*mug61*^+^	LEM domain protein, Sad1 interacting factor	HU, BLM, UV	2C	This study
SPAC1556.06^f^	*meu1*^+^	meiotic expression up-regulated protein	HU, BLM, UV	NC	This study
SPAC22E12.11c	*set3*^+^	histone lysine methyltransferase	BLM, MMS, UV	W4C	This study
SPAC25H1.05^f^	*meu29*^+^	meiotic expression up-regulated protein 29 precursor	HU, BLM, TBZ	W4C	This study
SPAC27D7.05c	*apc14*^+^	anaphase-promoting complex subunit	HU, TBZ, UV	NC	This study
SPAC3G6.01	*hrp3*^+^	ATP-dependent DNA helicase	BLM, TBZ, UV	2C	This study
SPBP8B7.13	*vac7*^+^	Vac7 ortholog	HU, MMS, TBZ	W4C	This study
SPCC830.06^d^		calcineurin regulatory subunit	HU, BLM, TBZ	W4C	This study
SPAC27D7.08c^e^		DUF890 family protein	HU, BLM	1C	This study
SPAC3F10.17^d^		ribosome biogenesis protein	HU, BLM	2C	This study
SPBC29A10.02	*mug12*^+^	meiotic RNA-binding protein	HU, BLM	NC	This study
SPBC31E1.02c	*pmr1*^+^	P-type ATPase, calcium transporting	HU, UV	NC	This study
SPBC577.13^d^	*syj2*^+^	inositol polyphosphate phosphatase	HU, TBZ	NC	This study
SPCC1494.03	*arz1*^+^	Zfs1 target number 1	MMS, UV	1C	This study
SPBC20F10.10^d^	*psl1*^+^	cyclin pho85 family	MMS	NC	This study

^a^ See text for detailed descriptions of flow cytometry phenotypes.

^b^ Reference that reported involvement of gene in DDR.

^c^ NC, no change.

^d^ role inferred from homology.

^e^ conserved hypothetical.

^f^ sequence orphan.

Among these 52 genes, 24 genes (46%) were identified in previous large-scale screens [12,13], and 32 genes (62%) in total have been reported to be related with DDR, which validates the accuracy of our screen (Table 1). For example, genes directly involved in sensing and repairing DNA damage were identified. Proteins encoded by these genes include: Rad1 and Rad9, two subunits of a checkpoint complex (9-1-1) [20]; Crb2, Rep2 and Ulp2, proteins required for cell cycle control [19,27,29]; Rhp55, Sen1 and Srs2, proteins involved in DNA double strand break (DSB) and single strand break (SSB) repair [22,25,33]. As expected, deletions of these genes were sensitive to a broad range of DNA damage reagents (Table 1). Genes involved in spindle assembly and cytokinesis were also obtained, including *dad5*^+^, *atb2*^+^, *mad1*^+^, *pab1*^+^, *myo1*^+^ and *scd1*^+^[24,26,28,31,32,36]. As expected, deletions of these genes exhibited sensitivity to TBZ, a microtubule depolymerizing agent (Table 1). Chromatin controls the accessibility of the DNA repair machinery, and thus it was not surprised to identify genes related to the dynamics of chromatin structure. Such proteins included Set1 and Ash2, subunits of a histone H3K4 methyltransferase complex [30]; Clr4 and Swi6, subunits of an H3K9 methyltransferase [35]; Gcn5, Sgf73 and Spt20, subunits of the SAGA histone acetylase complex [23]; Pst2, a component of Clr6 deacetylase complex [21]; Snf5, a subunit of the Swi/Snf remodeling complex [38]; Pht1, a histone H2A variant [34]. These results stress the importance of histone modification and chromatin remodeling in DDR. *SPBC409.15*, *sec65*^+^, *tcg1*^+^, *cch1*^+^ and *SPAC19A8.11c* were identified previously during other genome-wide screens [12,13]. Identification by our screen confirmed the relevance of these genes to DDR. However, several known DDR genes identified in the previous large scale screens, including *ctp1*^+^, *rhp51*^+^, *rad32*^+^, *rad26*^+^, *pnk1*^+^, *rad3*^+^, *hus1*^+^, *rad17*^+^, *rad24*^+^, *rhp57*^+^[12,13], were not screened out in this study. This might be caused by different screen strategy, different choice of DNA damaging agents and their working concentrations. Besides, the commercial library we used contained errors. We checked the mutants of several known DDR genes and found *rhp51*Δ, *rad26*Δ, *rad3*Δ were wrong. Therefore, the quality of the library also affected the results of our screen.

On the other hand, another 20 genes were found to be linked with DDR for the first time in this study, and the identities of corresponding mutants have been double checked. Among 20 genes, 10 genes have been already identified to function in different biological processes, including biosynthesis, RNA processing, stress response, transport and chromatin modification. Notably, deletion of *trk1*^+^, a gene encoding the potassium ion transporter, caused strong sensitivity to almost all the DNA damage reagents used in our assay [39]. There was no assigned function for the remaining 10 genes; they were classified as “sequence orphan”, “conserved hypothetical” or “role inferred from homolog”. Our data provided novel functional annotations for these unknown genes. Interestingly, deletion of *psl1*^+^ and *SPAC19A8.11c* caused sensitivity to only one reagent, suggesting these genes are required for repairing a specific DNA lesion.

Among these 20 novel DDR genes, 11 genes have homologues in *S. cerevisiae*. Notably, deletion of 5 homologous genes are sensitive to DNA damage reagents in *S. cerevisiae* (Table 2), which reflects the functional conservation of these DDR genes in fungi [40-44].

**Table 2:** List of homologues of novel DDR genes in *S. cerevisiae*

**Systematic ID in *S. pombe***	**Gene name in *S. pombe***	**Systematic ID in *S. cerevisiae***	**Gene name in *S. cerevisiae***	DNA damaging agents*	**Reference**
SPAC3F10.02c	*trk1*^+^	YJL129C	*TRK1*	-	
SPAC1486.04c	*alm1*^+^	YKR095W	*MLP1*	BLM, MMS, UV	[40-42]
SPAC17G6.06	*rps2401*^+^	YER074W	*RPS24A*	HU	[43]
SPBC2A9.02		YLL056C		-	
SPAC22E12.11c	*set3*^+^	YPL181W	*CTI6*	-	
SPAC3G6.01	*hrp3*^+^	YER164W	*CHD1*	HU	[43]
SPCC830.06		YKL190W	*CNB1*	-	
SPAC3F10.17		YKL143W	*LTV1*	-	
SPBC31E1.02c	*pmr1*^+^	YGL167C	*PMR1*	HU, TBZ	[43,44]
SPBC577.13	*syj2*^+^	YOR109W	*INP53*	BLM	[43]
SPBC20F10.10	*psl1*^+^	YIL050W	*PCL7*	-	

* Listed are the DNA damage reagents that deletion of *S. cerevisiae* genes are sensitive to.

### Cell cycle analysis of DNA damage sensitive mutants

**Figure 1:**
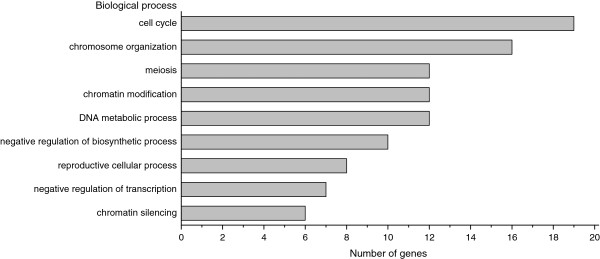
**Representative GO terms of 52 genes.** GO terms shown in the chart are: cell cycle (GO:0007049), chromosome organization (GO:0051276), meiosis (GO:0007126), chromatin modification (GO:0016568), DNA metabolic process (GO:0006259), negative regulation of biosynthetic process (GO:0009890), reproductive cellular process (GO:0048610), negative regulation of transcription (GO:0016481) and chromatin silencing (GO:0006342). Complete list of GO terms is shown in Additional file 1: Table S2.

*S. pombe* genome is extensively annotated using terms from the Gene Ontology Consortium (http://www.geneontology.org), with 98.3% of its genes having at least one GO (Gene Ontology) annotation [45]. The GO term classification of 52 genes was carried out with a significance level smaller than 0.05 (Additional file 1: Table S2), and representative GO terms were shown in Figure 1. This analysis revealed that the 52 genes were significantly enriched in cell cycle and chromatin related processes. As the most over-represented GO term, “cell cycle” was annotated to 36.5% of genes (19/52). Cell cycle control is one of the essential components of the DDR network [46,47]. After DNA damage, the cell cycle is delayed by checkpoint to provide an opportunity for repair. To monitor the cell cycle change in the deletions upon DNA damage, the DNA content of 52 mutants was analyzed by flow cytometry (Additional file 1: Table S3 and Figures S2-S5).

**Figure 2:**
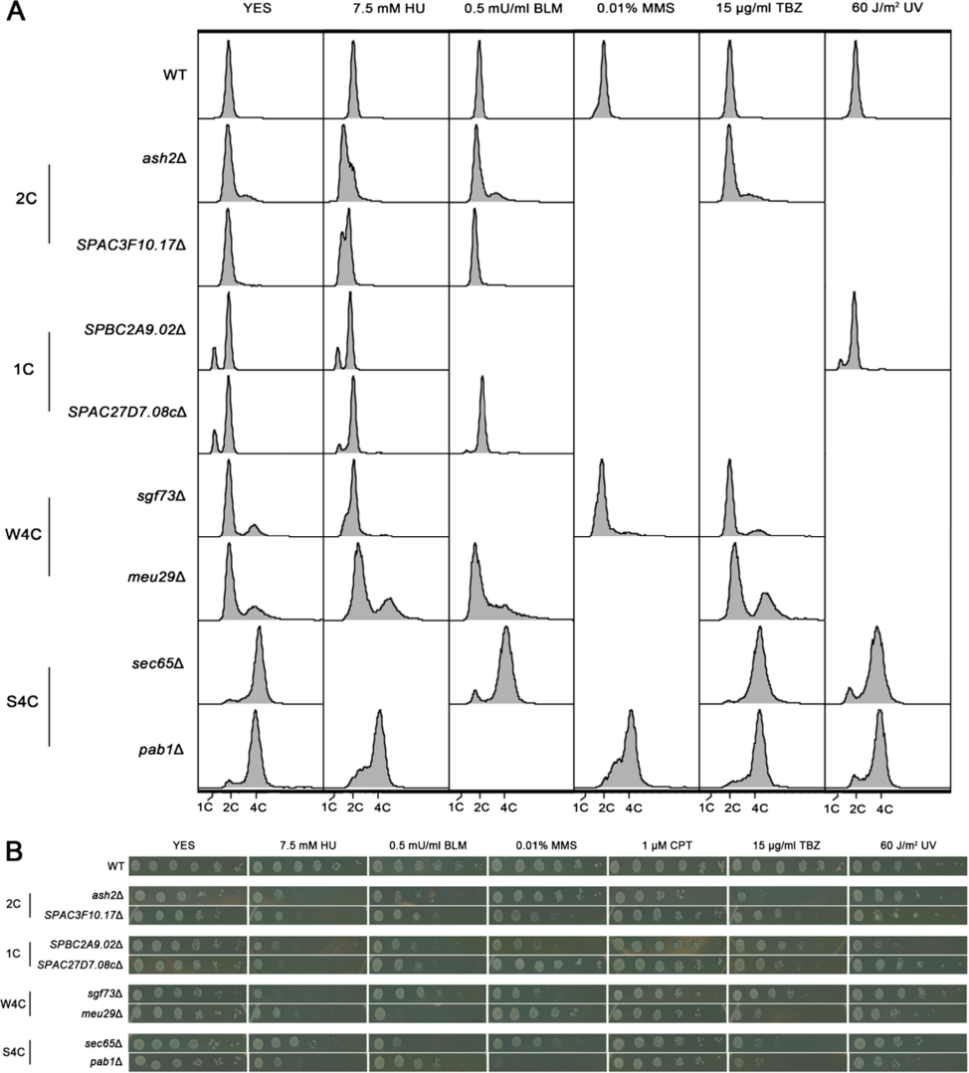
**Flow cytometry analysis and spot assays of eight representative mutants.** (**A**) Flow cytometry analysis of eight mutants. Cells were grown to the logarithmic phase and treated with a DNA damage reagent for 2 h. For UV sensitivity assay, cells were exposed to 60 J/m^2^ radiation and then grown for 2 h. After treatment cells were harvested and subjected to cytometry analysis. (**B**) Sensitivity to different DNA damage reagents was quantified by spot assays. Exponentially growing cells, WT or deletions, were harvested and 5-fold serial dilutions were spotted on plates supplemented with DNA damage reagents. The plates were photographed after 3~4 days of incubation at 32°C.

As expected, 37 deletions exhibited abnormal cell cycle profiles after DNA damage. No change was observed for the remaining 15 mutants, probably due to insufficient time for treatment. Based on flow cytometry phenotypes without reagent treatment, the 37 mutants could be divided into four groups which were designated as “2C”, “1C”, “W4C” and “S4C”, respectively (Table 1). Representative cytometry data of each group are shown in Figure 2A. “2C” stands for 2C DNA content. Members of this group, 16 deletions in total, exhibited DNA content peaks at 2C without reagent treatment, the same as WT cells. However, peaks moved towards 1C upon DNA damage caused by HU or MMS, suggesting that these deletions can cause replication arrest in response to damage (Additional file 1: Figure S2). The concentration of HU was the critical concentration that did not cause replication arrest of WT cells (Figure 2A). In the “1C” group, including 9 members, DNA content peaks moved towards 1C without treatment (Additional file 1: Figure S3). This result suggested that these deletions might have a defect in DNA replication [48,49]. Eight mutants in the “W4C” group and 4 mutants in the “S4C” group exhibited peaks of 4C DNA content (Additional file 1: Figure S4-S5) where “W” stands for “Weak”, as the 4C content was less than 35% and “S” represents “Strong”, because the 4C content was above 80%. Cytometry phenotypes suggested members of both groups had undergone diploidization, and the situation was much more severe in the “S4C” group. Genome duplication could be caused by DNA re-replication, a chromosome segregation defect, or improper cytokinesis [50]. Possible reasons for diploidization in the deletions will be discussed in the following section. Quantifications of the 1C, 2C and 4C DNA contents in 37 mutants are listed in Additional file 1: Table S3.

### Gene expression profiling of mutants

**Figure 3:**
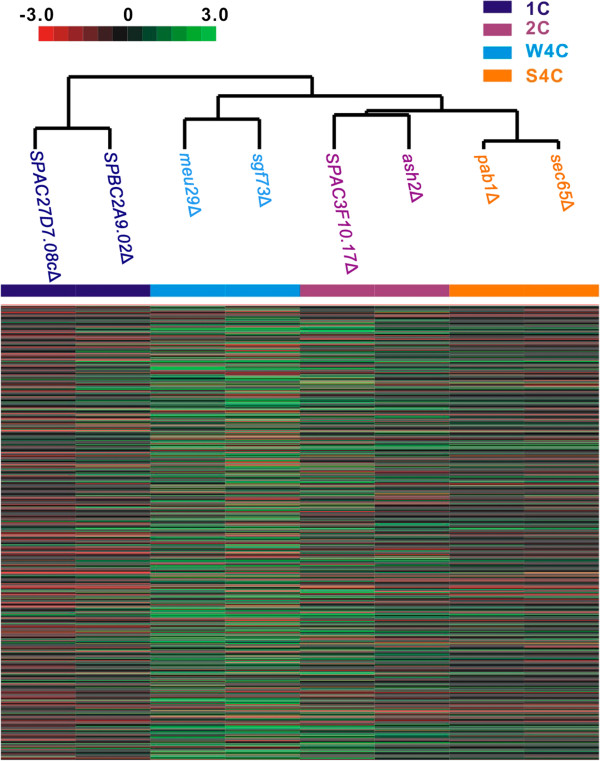
**Clustering analysis of eight mutants.** Hierarchical clustering matrix of eight mutants with microarray data for *S pombe* gene transcription. Color panel indicates relative increase (red) and decrease (green) to the median (black) of eight mutants for each transcript.

We selected 2 typical mutants from each cytometry phenotype group for further characterization (Figure 2A). All deletions showed strong sensitivity to at least two different DNA damage reagents (Figure 2B). *SPAC3F10.17*, *SPBC2A9.02*, *SPAC27D7.08c* and *meu29*^+^ were uncharacterized DDR genes. *ash2*^+^, *sgf73*^+^, *sec65*^+^ and *pab1*^+^ were identified during a previous global screen, but their detailed roles in DDR had not been identified yet [12,13]. For a better understanding of the gene function, we performed a DNA microarray assay to analyze the gene expression profiles of these eight deletions [51]. Transcription levels of hundreds of genes changed by 2-fold or more in the mutants. Notably, differentially regulated genes were enriched in the process related to DNA replication and cytokinesis. Representative genes are listed in Table 3. Analysis of microarray data by hierarchical clustering clustered 8 mutants into 4 groups (Figure 3). Notably, clustering perfectly matched the classification based on the flow cytometry phenotypes. It suggested that both genes from each group might function in the same pathway to regulate DDR and cell cycle progression.

**Table 3:** Differentially regulated genes in eight deletions

**Systematic ID**	**Gene name**	**Description of gene products**	**2C**		**1C**		**W4C**		**S4C**		**Gene ontology**
			***ash2*Δ**	***SPAC3F10.17*Δ**	***SPBC2A9.02*Δ**	***SPAC27D7.08c*Δ**	***sgf73*Δ**	***meu29*Δ**	***sec65*Δ**	***pab1*Δ**	
SPAC14C4.09	*agn1*^+^	glucan endo-1,3-alpha-glucosidase	2.7	2.0	2.4	1.9	2.6	1.9	2.9	3.1	cell septum edging catabolic process
SPAC6G10.12c	*ace2*^+^	transcription factor	4.1	3.8	1.3	1.4	2.4	1.6	3.9	3.4	cytokinetic cell separation
SPAC821.09	*eng1*^+^	endo-1,3-beta-glucanase	5.7	4.1	1.5	1.1	3.1	4.2	6.3	5.9	primary cell septum disassembly
SPBC83.18c	*fic1*^+^	C2 domain protein	1.8	1.6	1.3	1.3	2.3	1.9	1.3	1.3	cell cycle cytokinesis
SPCC320.13c	*ark1*^+^	aurora-B kinase	1.4	1.4	1.2	1.5	2.7	2.3	1.5	1.3	cell cycle cytokinesis
SPAC17H9.19c	*cdt2*^+^	WD repeat protein	2.6	1.4	3.3	1.6	2.5	1.2	2.2	3.0	DNA replication checkpoint
SPAC1F7.05	*cdc22*^+^	ribonucleoside reductase large subunit	2.7	2.3	2.1	1.6	3.3	2.3	2.5	2.8	regulation of DNA-dependent DNA replication
SPAC27E2.10c	*rfc3*^+^	DNA replication factor C complex subunit	1.7	1.2	1.1	1.1	1.6	2.1	1.3	1.3	DNA-dependent DNA replication
SPAC3G6.06c	*rad2*^+^	FEN-1 endonuclease	0.98	1.8	1.6	1.6	2.1	2.5	1.7	1.7	DNA replication, removal of RNA primer
SPAC821.08c	*slp1*^+^	sleepy homolog	2.6	2.2	1.2	1.2	3.6	2.0	2.5	2.2	DNA replication checkpoint
SPBC1105.04c	*abp1*^+^	CENP-B homolog	0.64	0.69	0.43	0.40	0.41	0.46	0.57	0.52	DNA-dependent DNA replication initiation
SPBC12D12.02c	*cdm1*^+^	DNA polymerase delta subunit	1.9	2.2	1.5	1.9	1.9	1.3	2.8	3.1	DNA strand elongation involved in DNA replication
SPBC14C8.07c	*cdc18*^+^	MCM loader	1.9	1.4	1.4	1.0	2.3	1.2	2.0	1.9	DNA replication checkpoint
SPBC1861.02	*abp2*^+^	ARS binding protein	1.5	1.2	0.28	0.50	0.31	0.70	0.91	0.80	DNA-dependent DNA replication initiation
SPBC428.18	*cdt1*^+^	replication licensing factor	3.0	2.8	2.3	1.7	2.2	2.2	3.5	3.3	DNA replication checkpoint
SPBC660.14	*mik1*^+^	mitotic inhibitor kinase	2.5	3.2	1.5	1.5	3.4	1.7	2.3	2.4	DNA replication checkpoint
SPCC1672.02c	*sap1*^+^	switch-activating protein	1.2	0.75	1.6	1.0	2.3	1.2	1.4	1.4	replication fork arrest at rDNA repeats
SPCC23B6.05c	*ssb3*^+^	DNA replication factor A subunit	2.1	1.8	1.1	1.6	1.3	1.4	1.2	1.3	DNA-dependent DNA replication
SPCC970.10c	*brl2*^+^	ubiquitin-protein ligase E3	2.6	1.3	1.1	0.89	1.1	1.2	1.1	1.1	DNA replication-independent nucleosome assembly

Differentially regulated genes in the deletions comparing to WT were analyzed using an Affymetrix microarray. Genes relevant to DNA replication and cytokinesis are listed. The number represents the ratio of mRNA level in the deletion relative to that in WT.

### *abp1*^+^ and *abp2*^+^ function downstream of *SPBC2A9.02* and *SPAC27D7.08c* to initiate DNA replication

**Figure 4:**
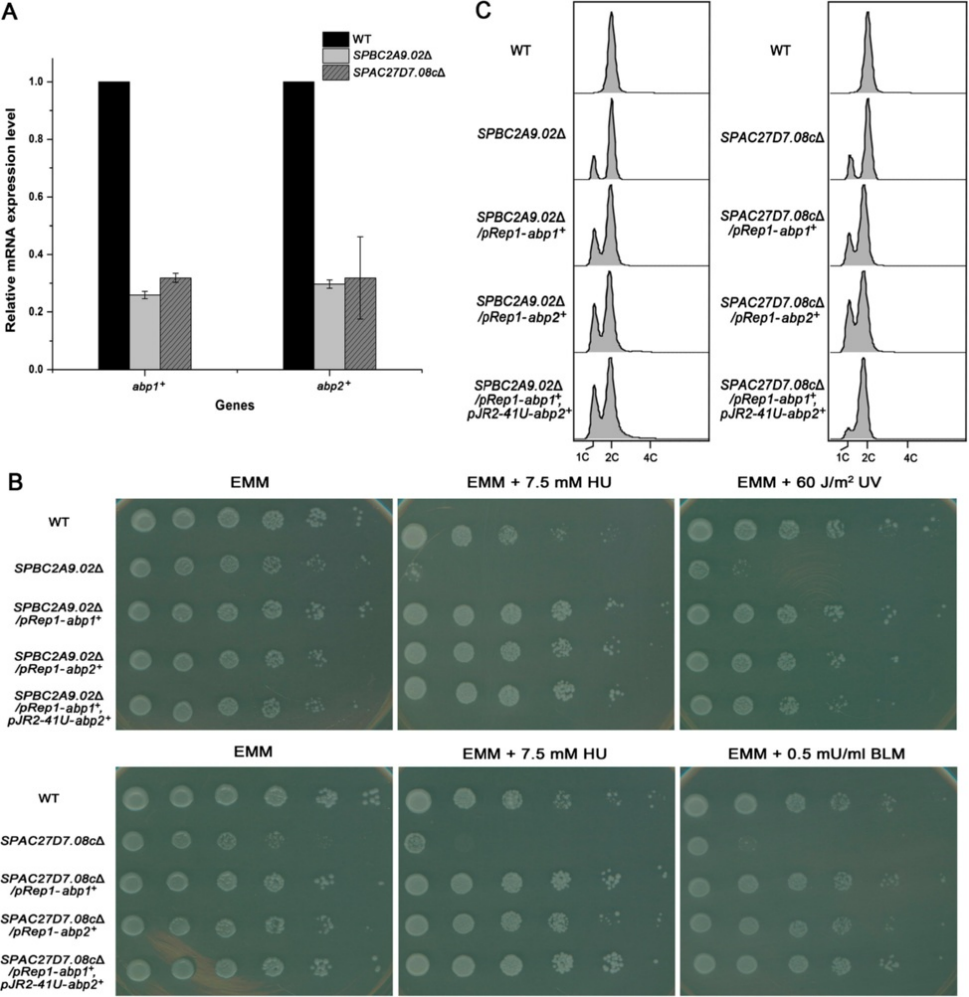
***abp1*^**+ **^and *abp2*^**+ **^function downstream of *SPBC2A9.02 *and *SPAC27D7.08c *to initiate DNA replication.** (**A**) Reduced expression levels of *abp1*^+^ and *abp2*^+^ in *SPBC2A9.02*Δ and *SPAC27D7.08c*Δ. The mRNA levels were quantified by real time PCR and those of *act1*^+^ served as an internal control (n=3). The relative level in WT was designated as arbitrary unit 1. (**B**) Overexpression of *abp1*^+^ and *abp2*^+^ partially rescued the growth defect of *SPBC2A9.02*Δ and *SPAC27D7.08c*Δ. *pREP1-abp1*^+^ or *pREP1-abp2*^+^ were transformed into each deletion separately. *pREP1-abp1*^+^ and *pJR2-41U-abp2*^+^ were co-transformed into *SPBC2A9.02*Δ or *SPAC27D7.08c*Δ. Transformants were harvested and 5-fold serial dilutions were spotted on plates supplemented with DNA damage reagents. Plates were photographed after 3 days of incubation at 32°C. (**C**) Overexpression of *abp1*^+^ or *abp2*^+^ partially relieved the G1-arrest in *SPBC2A9.02*Δ and *SPAC27D7.08c*Δ. Transformants described in Figure 4B were grown to logarithmic phase and harvested for flow cytometry analysis. Reproducible results were obtained in three independent experiments.

As members of the “1C” group, *SPBC2A9.02*Δ or *SPAC27D7.08c*Δ exhibited a discrete 1C DNA peak, suggesting G1 arrest and a defect in replication initiation [52]. Consistently, both mutants displayed a growth defect on EMM plates (Figure 4B). Both microarray and real-time PCR analysis revealed that the expression levels of *abp1*^+^ and *abp2*^+^ were simultaneously down-regulated by more than 2-fold in both deletions (Table 3 and Figure 4A). Abp1 and Abp2 are ARS (autonomously replicating sequence) binding proteins and are required for initiation of DNA replication [53,54]. It is possible that down-regulation of *abp1*^+^ and *abp2*^+^ contributed to the replication defects observed in *SPBC2A9.02*Δ and *SPAC27D7.08c*Δ. To check this possibility, we overexpressed *abp1*^+^ and *abp2*^+^ in the deletions. Without DNA damage, the growth defects of *SPBC2A9.02*Δ and *SPAC27D7.08c*Δ were partially rescued by overexpression of *abp1*^+^ and *abp2*^+^ (Figure 4B). The improvement was more obvious in the case of *SPAC27D7.08c*Δ, and was relatively mild, nevertheless, observable in the case of *SPBC2A9.02*Δ. In face of DNA damage, overexpressing either *abp1*^+^ and *abp2*^+^ could significantly improve the growth of *SPBC2A9.02*Δ and *SPAC27D7.08c*Δ (Figure 4B). Correspondingly, G1-arrest in *SPAC27D7.08c*Δ could also be reproducibly relieved by overexpression of both *abp1*^+^ and *abp2*^+^ (Figure 4C). The data suggested that *abp1*^+^ and *abp2*^+^ function downstream of *SPBC2A9.02* and *SPAC27D7.08c* to ensure the proper initiation of DNA replication under normal circumstances or after DNA damage.

### Members of “W4C” and “S4C” groups exhibited defects in cytokinesis and replication

**Figure 5:**
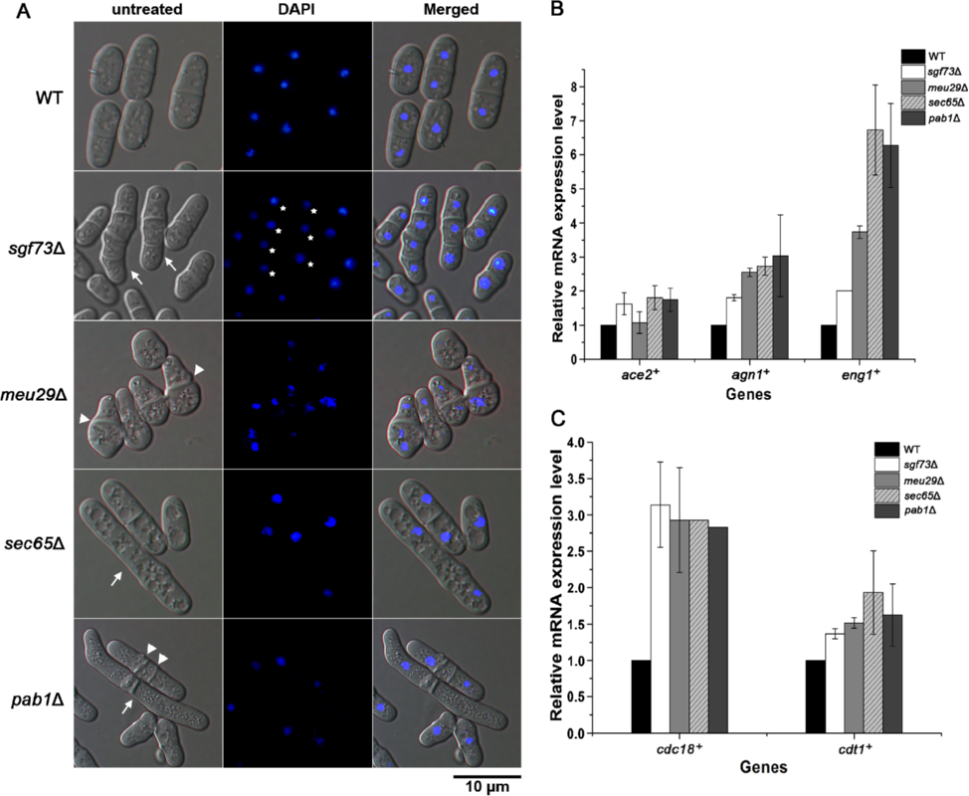
***sgf73*^**+**^, *meu29*^**+**^, *sec65*^**+ **^and *pab1*^**+ **^function in cytokinesis and DNA replication.** (**A**) Microscopic analysis of deletions. Cells were stained with DAPI to visualize the nuclei. Cells exhibiting elongated cell length are indicated by arrows, multiple nuclei by asterisk, and abnormal septum by arrow heads. The bar represents 10 μm. (**B**) Increased expression levels of *ace2*^+^, *agn1*^+^ and *eng1*^+^ in the deletions. The mRNA levels were quantified by real time PCR and those of *act1*^+^ served as internal controls (n=3). The relative level in WT was designated as arbitrary unit 1. (**C**) Increased expression levels of *cdc18*^+^ and *cdt1*^+^ in deletions. Real time PCR was performed as described in Figure 5B (n=3).

Deletions from the “W4C” and “S4C” groups exhibited discrete peaks of 4C DNA content, suggesting the mutants underwent diploidization. Diploidization in *S. pombe* is commonly caused by a defect in cytokinesis. Correspondingly, microscopic analysis revealed abnormal morphological changes in these mutants (Figure 5A). WT cells were rod-shaped and contained a single nucleus, or double nuclei separated by a sharp septum. In contrast, mutant cells exhibited elongated cell length (*sgf73*Δ, *sec65*Δ and *pab1*Δ), multiple nuclei (*sgf73*Δ), thick septum (*meu29*Δ) or multiple septa (*pab1*Δ), resembling typical defects in cytokinesis [55]. As expected, all 4 deletions displayed strong sensitivity to TBZ, a microtubule depolymerizing agent [56]. Microarray and real-time PCR analysis showed that the expressions of several cytokinesis related genes were up-regulated in the mutants, including those of *ace2*^+^, *agn1*^+^ and *eng1*^+^ (Table 3 and Figure 5B). Ace2 is a transcription factor that controls the expression of genes required for cell separation, while *eng1*^+^ and *agn1*^+^ are both targets of Ace2. Eng1, a β-glucanase, degrades the primary division septum between the new ends of daughter cells. Agn1, an α-glucanase, hydrolyses the old cell wall surrounding the septum and leads to full separation of daughter cells [57,58]. The data suggest that deletion of *sgf73*^+^, *meu29*^+^, *sec65*^+^ or *pab1*^+^ delays proper progression of cytokinesis, while a ruptured cell wall constitutively generates a signal to activate the Ace2 pathway and up-regulate target genes [58].

On the other hand, diploidization could also result from DNA re-replication during one cell cycle. Consistent with this idea, expression levels of *cdc18*^+^ and *cdt1*^+^ were up-regulated in all 4 mutants (Table 3 and Figure 5C). Presence of Cdc18 and Cdt1 at pre-RCs (pre-replicative complexes) is important for efficient DNA replication initiation, and inactivation of these proteins after initiation is crucial to ensure only one round of DNA replication in each cell cycle. Overexpression of *cdc18*^+^ and *cdt1*^+^ in fission yeast causes replication origins to re-fire, and drive re-replication of DNA sequences genome-wide [59,60]. Therefore, up-regulation of *cdc18*^+^ and *cdt1*^+^ in *sgf73*Δ, *meu29*Δ, *sec65*Δ and *pab1*Δ might lead to DNA re-replication, and that contributes to the observed diploidization. Meanwhile, disturbed replication initiation probably compromises DDR during early S phase. Correspondingly, all the members in “W4C” and “S4C” groups showed strong sensitivities to HU.

## Discussion

In this study, six reagents were applied in the screen and they can cause different kinds of DNA damage. The screen revealed six genes whose deletions displayed strong sensitivities to five reagents, including *rad1*^+^, *rhp55*^+^, *ulp2*^+^, *pst2*^+^, *mlo3*^+^ and *trk1*^+^ (Table 1). Broad sensitivities to different DNA damage reagents suggest that these genes function in the universal pathway of DDR, for example, in the conserved ATM/ATR pathway [2]. As expected, *rad1*^+^ plays a role in the ATR pathway, and *rhp55*^+^ in the ATM pathway [2,22]. *ulp2*^+^, encoding a SUMO protease, is required for cell division after termination of the DNA damage checkpoint [61]. The high sensitivity of *ulp2*Δ to a broad range of DNA damage reagents emphasizes the importance of silencing of the DNA damage checkpoint and restart of the cell cycle. *pst2*^+^ encodes a subunit of the Clr6 histone deacetylase. Deletion of *pst2*^+^ could lead to hyperacetylation of histones and down-regulation of histone H3 S10 phosphorylation, resulting in abnormal chromosome condensation and a defect in DNA damage repair [62]. Identification of *pst2*^+^ during the screen indicates the importance of chromatin condensation and decondensation in DDR. The protein encoded by *mlo3*^+^ was required for export and quality control of mRNA [63], suggesting DDR is related to the level and quality of mRNA. The screen has revealed the novel link between DDR and *trk1*^+^, gene encoding a potassium ion transporter [39]. Two calcium transporter genes, *cch1*^+^, and *pmr1*^+^, have also been identified in this study. *cch1*^+^, along with other ion transporter genes, including *zrg17*^+^, *fep1*^+^, *ctr4*^+^ and *zhf1*^+^, were identified during previous global screens for DDR genes [12,13].These results imply a close connection between ion transport and DDR. Ion transport controls several crucial physiological parameters, including membrane potential and ion balance [64]. It will be intriguing to uncover the mechanism how ion transport influences the DDR in future studies.

The screen also identified genes whose deletion exhibited sensitivity to only one kind of DNA damage reagent. Characterization of these genes will help to elucidate the specific DDR for a certain DNA lesion. For example, deletion of *psl1*^+^ displayed specific sensitivity to MMS. Previous screens have identified similar genes, including *cac2*^+^, *mag1*^+^, *rev3*^+^ and *slx4*^+^[9]. These genes, along with *psl1*^+^, might work together to remove the damage caused by alkylated DNA. *SPAC19A8.11c*Δ caused exclusive sensitivity to BLM. BLM abstracts a hydrogen atom from DNA deoxyribose and causes alkali-labile sites in DNA [15]. Genomic screen in budding yeast identified 23 genes exhibiting specific sensitivity to BLM [11]. *SPAC19A8.11c* might be an additional gene needed to repair lesions caused by BLM.

Cell cycle is delayed by checkpoints in response to DNA damage, thus providing a chance to repair DNA lesions. Several DNA damage checkpoints have been described in *S. pombe*, including G2-M, intra-S, S-M, G1-M and G1-S checkpoints [46,65-68]. Among the 52 deletion identified in this study, 37 deletions were found to affect cell cycle progression. Notably, 16 deletions in the “2C” group caused replication arrest upon treatment with HU or MMS. It suggested that these genes might be involved in DNA damage repair in S phase. Failures of repairing lesions in the deletions might persist intra-S checkpoint and slow the replication. Another member of “2C”, *myo1*Δ caused a 4C peak of DNA content after treatment of TBZ, indicating the diploidization of the genome. Since Myo1 regulates the assembly of actin and contributes to proper septation, observed diploidiation might be caused by a cytokinesis defect in *myo1*Δ [26].

In contrast to the “2C” group, deletions in the “1C” group caused G1 or S phase arrest without DNA damage. The data suggest these genes are required for cell cycle progression. These deletions interfere with cell cycle regulation in response to DNA damage, thus leading to high sensitivity to damage reagents. Further investigation revealed that SPBC2A9.02 and SPAC27D7.08c might function in the initiation of DNA replication through initiation factors, Abp1 and Abp2. Since deletion of *SPBC2A9.02* and *SPAC27D7.08c* share a similar cytometry phenotype and gene expression profiling, it is likely both genes work in the same pathway. SPAC27D7.08c contains a methyltransferase 10 domain, harboring potential SAM-dependent methyltransferase activity (http://www.rcsb.org/pdb/explore.do?structureId=2H00). It suggests that SPAC27D7.08c might regulate replication by methylating downstream proteins.

Flow cytometry analysis indicated that the members of “S4C” and “W4C” groups underwent diploidization. Gene expression and microscopic analysis of *sgf73*Δ, *meu29*Δ, *sec65*Δ and *pab1*Δ suggested diploidization might be caused by a cytokinesis defect and DNA re-replication. It is possible that proteins encoded by these genes function as subunits of large complexes, involved in the regulations of different processes, including replication, chromosome segregation and cytokinesis. A similar case was reported for a subunit of the Orc complex, Orc6 [69]. Consistent with this idea, Sgf73 is a subunit of the SAGA complex, a conserved multifunctional co-activator [70]. SAGA complex is known to regulate transcriptional activation, transcription elongation and mRNA export [71]. However, its roles in DNA re-replication and cytokinesis are yet to be identified. Recently, Pab1 has been revealed to be a novel component of the septation initiation network (SIN) complex [24]. SIN plays an important role in cytokinesis [72]. Whether the SIN complex also contributes to the replication initiation needs further characterization. Notably, *pab1*^+^, along with other 3 genes from the “W4C” group (*SPCC830.06*, *set3*^+^, *atb2*^+^), is conserved from *S. pombe* to mammals. Thus, further characterization of these genes is expected to provide valuable information for studies of genome stability and DDR in higher eukaryotes, especially in human.

## Conclusions

**Figure 6:**
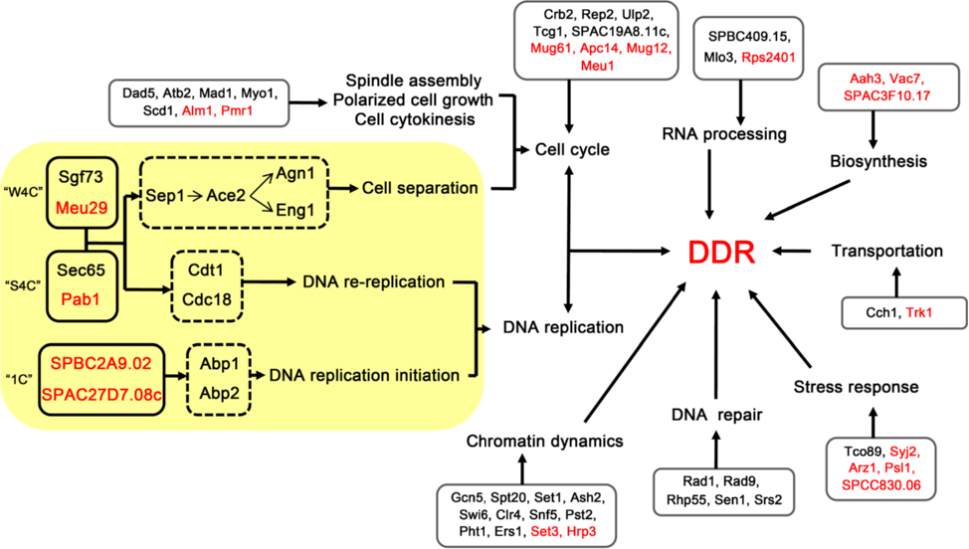
**Graphic presentation of the involvement of 52 genes in DDR network.** Proteins encoded by 52 DDR genes are shown in the solid line box and previously uncharacterized proteins are labelled in red. Proteins are categorized into different biological processes based on the GO analysis. Possible pathways that link 6 novel DDR proteins are shown in orange background.

Genome-wide screening is a fast and efficient way to explore unknown genes, clarify signaling pathways, and to ultimately build a comprehensive gene network. In this study, we performed a systematic screen of the *S. pombe* deletion library to uncover genes involved in DDR. 52 genes were characterized, among which 20 genes were linked to DDR for the first time. Most of the genes take part in cell cycle control, DNA repair, chromatin dynamics and DNA replication, all of which are well-known components of DDR [2]. In addition, many novel genes functioning in biosynthesis, transport, RNA processing and stress response were uncovered, suggesting their substantial contributions to DDR. Further characterizations suggested 6 novel genes might function in DDR through DNA replication and cytokinesis. Our study introduces new members to the long list of DDR genes and provides new clues to clarify the dynamic DDR network (Figure 6).

## Methods

### Genome-wide haploid deletion library

The *S. pombe* haploid deletion library used in this study was bought from Bioneer (http://pombe.bioneer.co.kr/). It contains 3,235 haploid deletion strains covering 65.8% of the 4,914 protein coding open reading frames (ORFs) based on the annotated genome sequence (http://www.genedb.org/genedb/pombe). As 3,576 genes are nonessential [73], this library represents approximately 90.5% of the nonessential *S. pombe* genes. Fission yeast were cultured in YES or EMM medium at 32°C as described before [74].

### Screen of deletions sensitive to DNA damage

The screen was performed in three rounds. In the first round, deletion strains from the Bioneer library were grown in YES medium till saturation. 20 μl culture from each strain was diluted into 180 μl liquid YES medium containing different DNA damage reagents in 96-well microtiter plates. As a control, cells were also diluted into medium without any reagent. Concentrations of reagents were: 7.5 mM hydroxyurea (HU) (SIGMA, H8627), 0.5 mU/ml bleomycin (BLM) (NIPPON KAYAKU, 480890), 0.01% methyl methanesulfonate (MMS) (ACROS ORGANICS, 156890050), 1 μM camptothecin (CPT) (SIGMA, C9911), 15 μg/ml thiabendazole (TBZ) (TCI, T0830) and 60 J/m^2^ ultraviolet radiation (UV). After 24 hours of incubation at 32°C, the optical densities of the cultures were measured at 600 nm (*A*_600_) and compared to those of the controls. Deletions with *A*_600_ that dropped by 5 fold or more upon reagent treatment were designated as sensitive. Deletion mutants showing sensitivity to at least one reagent were picked to create a sub-library. This round of the screen was repeated once. In the second round, strains from the sub-library were grown in YES medium overnight, and then inoculated into 1 ml YES medium containing different reagents at an *A*_600_ of 0.02. After 24 hours of incubation at 32°C, *A*_600_ was measured and compared to those of no reagent controls. In the third round, strains showing sensitivity to at least one DNA-damaging agent in the second round were grown in liquid medium to an *A*_600_ of 1.0. Cultures were diluted by five-fold for five times, and 2 μl dilutions were spotted onto YES or EMM plates containing DNA damage reagents of indicated concentrations. The growth of the cells was checked after 3~4 days of incubation at 32°C. If the growth of a mutant on the plate containing certain reagent was 2-spot lesser than that on YES plate (25 fold reduction in viability), this mutant was designated as sensitive.

### Gene ontology analysis

Gene ontology (GO) classifications were performed at http://amigo.geneontology.org with the database filter set as GeneDB *S. pombe*. Maximum *P*-value was 0.05 as the threshold for significance assessment, and minimum number of gene products was 3 in each GO term. GO analysis was based on the biological process classifications in this study [73].

### Flow cytometry

1~2×10^7^ exponentially growing cells were treated with DNA damage reagent for 2 h. For the UV sensitivity assay, cells were exposed to 60 J/m^2^ radiation and then grown for 2 h. Cells were harvested and fixed in 70% (v/v) cold ethanol at 4°C for 1 h. Cells were resuspended in 0.5 ml of 50 mM sodium citrate containing 0.1 mg/ml RNase A and incubated at 37°C for 2 h. Cells were briefly sonicated, and then stained with 4 μg/ml propidium iodide (PI) at room temperature for 15 min. 1~2×10^4^ cells were measured by a FACS Calibur flow cytometer (Becton-Dickinson) and data were analyzed by Flowjo 2.0 [48].

### DNA microarray analysis

cDNAs were prepared from the exponentially growing wild type (WT) cells or deletion cells as previously described [75]. cDNA was labeled and hybridized to the Yeast genome 2.0 array according to the manufacturer’s protocol (900553, Affymetrix). Data was analyzed by Shanghai GeneTech Company (Shanghai, China). The data discussed in this publication have been deposited in NCBI's Gene Expression Omnibus [76] and are accessible through GEO Series accession number GSE40747 (http://www.ncbi.nlm.nih.gov/geo/query/acc.cgi?acc=GSE40747).

### Clustering analysis

Hierarchical clustering was carried out by Gene Cluster with differentially regulated genes of eight mutants, using the correlation (uncentered) and centroid linkage clustering method. The clustering results were visualized with Java TreeView.

### Real time PCR analysis

Experiments were performed as described before [77]. Briefly, total RNAs were prepared from exponentially growing cells by using TRIzol (Invitrogen) and reverse-transcribed to make first strand cDNAs. cDNAs were used as templates for real time PCR. PCR were performed using SYBR Premix ExTaq TMII (DRR081C, Takara) on an ABI Prism 5700 sequence detection system (Applied Biosystems) according to manufacturer’s protocol. The threshold cycle (*C*_T_) of each sample was determined by the ABI system and then normalized to the value for *act1*^+^ by the following equation: Δ*C*_T_ = *C*_T(gene of interest)_ − *C*_T (*act1*+)_. Relative level was calculated as 2^-Δ*C*T^. Reaction for each sample was performed in triplicate. Primers are listed in Additional file 1: Table S4.

### Microscopic analysis

After overnight incubation at 32°C, cells were washed with phosphate-buffered saline and stained with 1 μg/ml 4’, 6’-diamidino-2-phenylindole (DAPI) to visualize nuclei. Cells were observed and captured by a Zeiss Axioplan microscope equipped with a chilled video charge-coupled device camera (C4742-95; Hamamatsu Photonics, Bridgewater, NJ). Images were analyzed by kinetic image AQM software (Kinetic Imaging, Nottingham, UK).

## Abbreviations

The abbreviations used are: DDR: DNA damage response; GO: Gene ontology; SAGA: Spt-Ada-Gcn5 acetyltransferase; HU: Hydroxyurea; BLM: Bleomycin; MMS: Methyl methanesulfonate; CPT: Camptothecin; TBZ: Thiabendazole; UV: Ultraviolet radiation; WT: Wild type; DSB: Double strand breaks; SSB: Single strand breaks.

## Competing interests

The authors declare that they have no competing financial interests.

## Authors’ contributions

XP carried out the screening work, performed the molecular genetic studies, analyzed the data, and drafted the manuscript. BL participated in the screening work and molecular genetic studies. NZ participated in the molecular genetic studies. BF and WY participated in the screening work. XZ conceived of the study. YY participated in the design of the experiments and helped to draft the manuscript. HL conceived the study, designed and coordinated the work, and critically revised the manuscript. All authors read and approved the final manuscript.

## Supplementary Material

Additional file 1 **Table S1.** List of genes whose deletions exhibited sensitivity to DNA damage reagents during the second round of screen. **Table S2.** GO profiling of 52 genes whose deletion mutants showed strong sensitivity to DNA damage reagents (*P* ≤ 0.05). **Table S3.** Flow cytometry analysis of 37 mutants. **Table S4.** Primers used for real time PCR analysis in this study. **Figure S1.** Spot assay of 52 deletions. Exponentially growing cells, WT or deletions, were harvested and 5-fold serial dilutions were spotted on the plates supplemented with DNA damage reagents. The plates were photographed after 3~4 days of incubation at 32°C. **Figure S2.** Flow cytometry analysis of deletions in “2C” group. **Figure S3.** Flow cytometry analysis of deletions in “1C” group. **Figure S4.** Flow cytometry analysis of deletions in “W4C” group. **Figure S5.** Flow cytometry analysis of deletions in “S4C” group. Click here for file
