# Supplementary material for: Identification of novel genes involved in DNA damage response by screening a genome-wide Schizosaccharomyces pombe deletion library
Source: BMC Genomics. 2012 Nov 23;13:662. doi: 10.1186/1471-2164-13-662 (PMC3536581; doi:10.1186/1471-2164-13-662)

**Supplemental Tables and Figures**

### Table S1 – List of genes whose deletions exhibited sensitivity to DNA damage reagents during the second round of screen.

| Systematic ID | Gene name | DNA damaging agents | Systematic ID | Gene name | DNA damaging agents |
| --- | --- | --- | --- | --- | --- |
| SPAC1093.01 |  | HU, TBZ | SPAPB8E5.04c |  | HU |
| SPAC110.01 | *ppk1*^+^ | HU | SPBC1105.13c |  | HU |
| SPAC110.02^*^ | *pds5*^+^ | HU, MMS | SPBC119.05c |  | HU |
| SPAC11D3.05 | *msf2*^+^ | MMS | SPBC1198.08 |  | HU |
| SPAC11E3.10 |  | MMS | SPBC1198.14c | *fbp1*^+^ | MMS |
| SPAC11G7.02^*^ | *pub1*^+^ | HU, CPT, TBZ | SPBC11B10.10c | *pht1*^+^ | HU, BLM, MMS, UV |
| SPAC12B10.11 | *exg2*^+^ | HU | SPBC11G11.01 | *fis1*^+^ | HU |
| SPAC12B10.16c | *mug157*^+^ | HU | SPBC11G11.03 | *mrt4*^+^ | HU |
| SPAC12G12.09 |  | HU | SPBC1289.10c | *adn2*^+^ | HU, MMS |
| SPAC12G12.12^*^ |  | MMS | SPBC12C2.01c |  | HU |
| SPAC139.02c | *oac1*^+^ | HU | SPBC1347.02 | *fkbp39*^+^ | HU |
| SPAC13A11.01c | *rga8*^+^ | HU | SPBC1347.07 | *rex2*^+^ | HU |
| SPAC13A11.05 |  | HU | SPBC1348.02 |  | HU |
| SPAC13D6.03c | *trm9*^+^ | HU | SPBC13E7.04 | *atp16*^+^ | HU |
| SPAC13G7.02c | *ssa1*^+^ | HU, MMS, TBZ | SPBC13G1.08c | *ash2*^+^ | HU, BLM, TBZ, UV |
| SPAC144.04c | *spe1*^+^ | HU, MMS, TBZ | SPBC13G1.10c | *mug81*^+^ | HU |
| SPAC1486.04c | *alm1*^+^ | HU, BLM, MMS, UV | SPBC146.13c^*^ | *myo1*^+^ | HU, BLM, TBZ, UV |
| SPAC14C4.05c | *mug61*^+^ | HU, BLM, UV | SPBC14F5.03c | *kap123*^+^ | HU, TBZ |
| SPAC14C4.12c | *laf1*^+^ | HU | SPBC1604.19c |  | HU |
| SPAC1527.01 | *mok11*^+^ | HU | SPBC1652.01 |  | HU, MMS |
| SPAC1556.06 | *meu1*^+^ | HU, BLM, UV | SPBC1683.11c |  | HU |
| SPAC15A10.03c^*^ | *rhp54*^+^ | HU, MMS, TBZ, UV | SPBC1685.14c |  | HU |
| SPAC15A10.06 |  | HU, MMS, TBZ | SPBC16C6.04 |  | HU |
| SPAC15F9.01c |  | HU | SPBC16D10.05 | *mok13*^+^ | HU |
| SPAC1610.02c^*^ |  | HU | SPBC16G5.03 |  | HU, MMS |
| SPAC1687.09 |  | HU, UV | SPBC16G5.07c |  | HU |
| SPAC1687.14c |  | HU | SPBC16H5.04 |  | HU, MMS |
| SPAC1687.19c |  | HU | SPBC16H5.12c |  | HU, TBZ |
| SPAC16E8.09 | *scd1*^+^ | HU, BLM, TBZ | SPBC1711.04 |  | HU, MMS |
| SPAC1782.11 | *met14*^+^ | HU, MMS | SPBC1711.12 |  | HU, MMS |
| SPAC1783.01 |  | HU | SPBC1711.13 | *his2*^+^ | HU |
| SPAC1783.06c | *atg12*^+^ | HU | SPBC1734.07c |  | HU, MMS |
| SPAC17A2.05 | *osm1*^+^ | HU, TBZ | SPBC1734.12c | *alg12*^+^ | HU, MMS |
| SPAC17A5.07c | *ulp2*^+^ | HU, BLM, MMS, TBZ, UV | SPBC1773.03c |  | HU |
| SPAC17A5.11 | *rec12*^+^ | HU, MMS | SPBC1778.02 | *rap1*^+^ | HU, MMS |
| SPAC17C9.10 | *stm1*^+^ | HU, MMS | SPBC1778.03c |  | HU |
| SPAC17G6.06 | *rps2401*^+^ | HU, BLM, MMS, UV | SPBC1778.09^*^ |  | HU |
| SPAC17G6.17 | *pof8*^+^ | HU, MMS | SPBC17D1.06 | *dbp3*^+^ | HU |
| SPAC17G8.14c | *pck1*^+^ | HU, MMS | SPBC17F3.01c | *rga5*^+^ | HU, MMS, TBZ |
| SPAC1805.02c |  | TBZ | SPBC1861.07 |  | HU |
| SPAC1805.05 | *cki3*^+^ | HU | SPBC18A7.01 |  | HU |
| SPAC1805.06c | *hem2*^+^ | HU | SPBC18H10.08c | *ubp4*^+^ | HU |
| SPAC1805.14^*^ |  | HU | SPBC19C2.10 |  | HU |
| SPAC186.01 |  | HU, MMS | SPBC19C7.01 |  | HU, CPT, TBZ |
| SPAC186.05c |  | HU | SPBC1D7.04 | *mlo3*^+^ | HU, BLM, MMS, CPT, UV |
| SPAC18B11.10 | *tup11*^+^ | HU | SPBC20F10.07^*^ |  | HU |
| SPAC1952.05^*^ | *gcn5*^+^ | HU, MMS, CPT, TBZ | SPBC20F10.10 | *psl1*^+^ | MMS |
| SPAC1952.07^*^ | *rad1*^+^ | HU, BLM, MMS, CPT, UV | SPBC215.01 |  | HU |
| SPAC1952.10c |  | HU | SPBC215.10 |  | HU, MMS |
| SPAC19A8.11c |  | HU, BLM, CPT, TBZ | SPBC215.14c | *vps20*^+^ | HU, MMS |
| SPAC19B12.08 | *atg4*^+^ | HU | SPBC21B10.07 |  | HU |
| SPAC19D5.03 | *cid1*^+^ | HU, MMS, TBZ | SPBC21B10.10^*^ | *rps402*^+^ | HU, MMS |
| SPAC19G12.08^*^ | *scs7*^+^ | HU | SPBC21C3.02c | *dep1*^+^ | HU, MMS |
| SPAC19G12.16c | *adg2*^+^ | HU | SPBC23E6.08^*^ | *sat1*^+^ | HU |
| SPAC1A6.05c |  | HU | SPBC23G7.04c | *nif1*^+^ | HU, MMS |
| SPAC1B2.04 | *cox6*^+^ | HU | SPBC25B2.04c | *mtg1*^+^ | HU |
| SPAC1B3.07c | *vps28*^+^ | HU | SPBC25B2.10 |  | HU |
| SPAC1B3.08 |  | HU | SPBC25H2.08c | *mrs2*^+^ | HU, MMS |
| SPAC1B3.16c | *vht1*^+^ | HU, TBZ | SPBC25H2.16c |  | HU, MMS |
| SPAC1D4.02c |  | HU | SPBC26H8.09c | *snf59*^+^ | HU |
| SPAC1F12.03c |  | HU | SPBC27B12.10c | *tom7*^+^ | HU, MMS |
| SPAC1F3.02c^*^ | *mkh1*^+^ | HU | SPBC29A10.02 | *spo5*^+^ | HU, BLM, MMS, TBZ |
| SPAC1F3.03 |  | HU | SPBC2A9.02 |  | HU, BLM, MMS, UV |
| SPAC1F3.09 | *mug161*^+^ | HU, MMS | SPBC2F12.03c |  | MMS |
| SPAC1F3.10c | *oct1*^+^ | HU | SPBC2F12.11c | *rep2*^+^ | HU, BLM, MMS, UV |
| SPAC20H4.02^*^ | *dsc3*^+^ | HU, MMS, TBZ | SPBC2F12.12c |  | HU |
| SPAC20H4.09 |  | HU, MMS | SPBC2G2.05 | *rpl1603*^+^ | HU, MMS, TBZ |
| SPAC212.02 |  | HU | SPBC2G2.08 | *ade9*^+^ | HU, MMS |
| SPAC212.04c |  | HU | SPBC30B4.06c |  | HU, MMS |
| SPAC227.07c^*^ | *pab1*^+^ | HU, MMS, TBZ, UV | SPBC30D10.09c |  | HU, MMS, TBZ |
| SPAC22A12.11 | *dak1*^+^ | HU | SPBC31E1.02c | *pmr1*^+^ | HU, UV |
| SPAC22E12.11c | *set3*^+^ | HU, BLM, MMS, UV | SPBC32H8.13c | *mok12*^+^ | HU |
| SPAC22F3.04 | *mug62*^+^ | HU | SPBC342.01c | *alg6*^+^ | HU, TBZ |
| SPAC22F8.04 |  | HU | SPBC342.05^*^ | *crb2*^+^ | HU, BLM, MMS, UV |
| SPAC22H10.13 | *zym1*^+^ | HU | SPBC354.01 | *gtp1*^+^ | HU, MMS |
| SPAC22H12.01c | *mug35*^+^ | MMS | SPBC365.01 |  | HU, MMS |
| SPAC22H12.03 |  | HU | SPBC365.12c | *ish1*^+^ | HU |
| SPAC23C11.15 | *pst2*^+^ | HU, BLM, MMS, CPT, TBZ, UV | SPBC3B9.08c |  | HU |
| SPAC23G3.12c |  | HU, MMS | SPBC3D6.04c | *mad1*^+^ | HU, BLM, TBZ, UV |
| SPAC23H3.15c |  | HU, MMS, TBZ | SPBC3D6.06c |  | HU |
| SPAC24C9.08 |  | HU, MMS, TBZ | SPBC3E7.02c | *hsp16*^+^ | HU |
| SPAC24H6.03^*^ | *cul3*^+^ | HU, BLM, TBZ | SPBC3H7.09 | *erf2*^+^ | HU, MMS |
| SPAC24H6.13 |  | HU, MMS | SPBC409.08 |  | HU |
| SPAC25B8.13c | *isp7*^+^ | HU | SPBC409.15 |  | HU, MMS, TBZ, UV |
| SPAC25B8.17 |  | HU | SPBC409.20c | *psh3*^+^ | HU, TBZ |
| SPAC25B8.18 |  | HU | SPBC428.08c^*^ | *clr4*^+^ | HU, MMS, TBZ |
| SPAC25H1.03 | *mug66*^+^ | HU, MMS | SPBC428.10 |  | MMS |
| SPAC25H1.05 | *meu29*^+^ | HU, BLM, TBZ | SPBC428.14 |  | HU |
| SPAC26A3.01 | *sxa1*^+^ | MMS | SPBC4B4.04 |  | HU, MMS, TBZ |
| SPAC26F1.12c |  | HU | SPBC4B4.06 | *vps25*^+^ | HU, MMS |
| SPAC27D7.05c | *apc14*^+^ | HU, TBZ, UV | SPBC4F6.12 | *pxl1*^+^ | HU, MMS, TBZ |
| SPAC27D7.08c |  | HU, BLM | SPBC530.01 | *gyp1*^+^ | HU, MMS |
| SPAC27F1.05c |  | MMS | SPBC530.08 |  | HU, MMS, TBZ |
| SPAC29B12.14c |  | HU, MMS, TBZ | SPBC56F2.03 |  | HU |
| SPAC2C4.17c |  | HU, MMS | SPBC577.13 | *syj2*^+^ | HU, TBZ |
| SPAC2E1P3.02c | *amt3*^+^ | HU, MMS | SPBC577.14c | *spa1*^+^ | HU, MMS, TBZ |
| SPAC2F3.08 | *sut1*^+^ | HU | SPBC660.06 |  | MMS |
| SPAC2F7.06c | *pol4*^+^ | HU | SPBC660.11 | *tcg1*^+^ | HU, BLM, MMS, TBZ |
| SPAC2F7.08c | *snf5*^+^ | HU, MMS | SPBC691.01 | *pfa5*^+^ | HU |
| SPAC2F7.10 | *akr1*^+^ | HU, MMS | SPBC713.11c | *pmp3*^+^ | HU |
| SPAC31A2.12 |  | HU | SPBC800.05c | *atb2*^+^ | HU, BLM, TBZ |
| SPAC31G5.11 | *pac2*^+^ | HU, MMS, TBZ | SPBC83.16c |  | HU |
| SPAC31G5.12c | *maf1*^+^ | HU, TBZ | SPBC8D2.04 | *hht2*^+^ | MMS |
| SPAC328.01c |  | HU, MMS | SPBC8D2.17 |  | HU |
| SPAC343.15 | *tit1*^+^ | HU | SPBC902.02c^*^ | *ctf18*^+^ | HU, MMS |
| SPAC3A11.04 |  | HU, MMS | SPBC947.01 |  | HU, MMS |
| SPAC3C7.03c^*^ | *rhp55*^+^ | HU, BLM, MMS, TBZ, UV | SPBP19A11.02c |  | HU, MMS |
| SPAC3F10.02c | *trk1*^+^ | HU, BLM, CPT, TBZ, UV | SPBP35G2.03c | *sgo1*^+^ | HU, MMS |
| SPAC3F10.07c | *erf4*^+^ | HU | SPBP4H10.09 | *rsv1*^+^ | HU, TBZ |
| SPAC3F10.17 |  | HU, BLM | SPBP8B7.09c | *los1*^+^ | HU |
| SPAC3G6.01 | *hrp3*^+^ | HU, BLM, TBZ, UV | SPBP8B7.11 | *nxt3*^+^ | HU |
| SPAC3H1.06c |  | HU | SPBP8B7.13 | *vac7*^+^ | HU, MMS, TBZ |
| SPAC4A8.04 | *isp6*^+^ | HU | SPBP8B7.18c |  | HU |
| SPAC4A8.05c | *myp2*^+^ | HU | SPBPB2B2.18 |  | HU, TBZ |
| SPAC4D7.10c | *spt20*^+^ | HU, MMS, CPT, TBZ, UV | SPBPB2B2.19c |  | HU, TBZ |
| SPAC4F8.01 | *did4*^+^ | HU | SPBPJ4664.06 | *gpt1*^+^ | HU, MMS, TBZ |
| SPAC4F8.11 |  | HU | SPCC1020.10 | *oca2*^+^ | HU, MMS, CPT, TBZ |
| SPAC4G8.08 |  | HU | SPCC11E10.04 |  | HU, MMS, TBZ, UV |
| SPAC4G9.13c | *vps26*^+^ | HU, BLM, MMS, TBZ | SPCC1223.09 |  | HU |
| SPAC4H3.05^*^ | *srs2*^+^ | HU, BLM, MMS, UV | SPCC1259.03 | *rpa12*^+^ | HU |
| SPAC56F8.14c | *mug115*^+^ | HU, MMS | SPCC1259.08^*^ |  | HU, MMS |
| SPAC57A10.09c | *nhp6*^+^ | HU, MMS | SPCC1259.11c | *gyp2*^+^ | HU, MMS |
| SPAC5H10.06c | *adh4*^+^ | HU, MMS | SPCC126.04c^*^ | *sgf73*^+^ | HU, MMS, TBZ |
| SPAC607.06c |  | HU, MMS | SPCC126.12 |  | MMS |
| SPAC637.10c | *rpn10*^+^ | HU, MMS | SPCC126.15c^*^ | *sec65*^+^ | HU, BLM, MMS, TBZ, UV |
| SPAC664.01c^*^ | *swi6*^+^ | HU, TBZ | SPCC1393.03 | *rps1501*^+^ | HU, MMS |
| SPAC664.07c^*^ | *rad9*^+^ | HU, BLM, MMS, UV | SPCC1393.05 | *ers1*^+^ | HU, BLM, TBZ, UV |
| SPAC688.13 | *scn1*^+^ | HU | SPCC1393.08 |  | HU, CPT, TBZ |
| SPAC688.14 | *set13*^+^ | HU, MMS | SPCC1494.03 | *arz1*^+^ | HU, MMS, UV |
| SPAC6F6.01^*^ | *cch1*^+^ | HU, MMS, UV | SPCC162.12 | *tco89*^+^ | HU, BLM, UV |
| SPAC6G10.11c | *ubi3*^+^ | HU, MMS | SPCC1620.13 |  | HU, MMS |
| SPAC6G9.10c | *sen1*^+^ | HU, BLM, MMS, TBZ, UV | SPCC1672.03c |  | HU, MMS, TBZ |
| SPAC6G9.15c |  | HU | SPCC1682.08c |  | HU |
| SPAC7D4.02c | *sfp47*^+^ | MMS | SPCC1682.15 | *mug122*^+^ | HU, BLM, MMS |
| SPAC823.05c | *tlg2*^+^ | HU, CPT, TBZ | SPCC1753.03c | *rec7*^+^ | HU |
| SPAC823.09c |  | MMS | SPCC1840.08c |  | HU |
| SPAC823.13c |  | HU, MMS | SPCC1919.01 | *ppk34*^+^ | HU |
| SPAC824.02^*^ |  | HU | SPCC306.04c | *set1*^+^ | HU, BLM, MMS, TBZ |
| SPAC824.09c |  | HU | SPCC338.14 |  | HU |
| SPAC869.04 |  | HU, MMS | SPCC338.16^*^ | *pof3*^+^ | HU, MMS |
| SPAC869.05c |  | HU, MMS, CPT | SPCC364.01 | *cif1*^+^ | HU, MMS |
| SPAC8C9.03^*^ | *cgs1*^+^ | HU, CPT, TBZ | SPCC364.04c |  | HU, MMS |
| SPAC8C9.04 |  | HU, MMS | SPCC417.02^*^ | *dad5*^+^ | HU, BLM, MMS, TBZ, UV |
| SPAC8C9.12c |  | HU, MMS, TBZ | SPCC4B3.03c |  | HU, MMS |
| SPAC922.03 |  | MMS | SPCC4G3.14 | *mdj1*^+^ | HU, MMS |
| SPAC926.07c | *dlc2*^+^ | HU, MMS | SPCC550.10 | *meu8*^+^ | HU, MMS |
| SPAC9E9.04 |  | HU | SPCC553.01c^*^ |  | HU, MMS |
| SPAC9E9.09c |  | HU, TBZ | SPCC584.02 | *cuf2*^+^ | HU, MMS |
| SPAC9G1.11c | *spn4*^+^ | HU | SPCC613.03 |  | HU, MMS |
| SPAC9G1.12 | *cpd1*^+^ | HU | SPCC622.08c | *hta1*^+^ | HU, MMS |
| SPACUNK4.16c |  | HU | SPCC622.15c |  | MMS |
| SPAP14E8.04 | *oma1*^+^ | HU, MMS | SPCC63.02c | *aah3*^+^ | HU, BLM, TBZ, UV |
| SPAP8A3.04c | *hsp9*^+^ | HU, MMS, TBZ | SPCC663.14c |  | HU |
| SPAP8A3.07c |  | HU, TBZ | SPCC737.09c | *hmt1*^+^ | HU |
| SPAP8A3.12c | *tpp2*^+^ | HU, MMS | SPCC74.06 | *mak3*^+^ | HU, MMS |
| SPAPB1A10.08 |  | HU, MMS | SPCC794.01c |  | HU, TBZ |
| SPAPB1A10.13 |  | HU, MMS | SPCC830.06 |  | HU, BLM, MMS, TBZ |
| SPAPB1A10.14 |  | HU, TBZ | SPCC895.09c | *ucp12*^+^ | HU |
| SPAPB1E7.02c | *mcl1*^+^ | HU, MMS, CPT, TBZ | SPCP1E11.05c^*^ | *are2*^+^ | MMS |
| SPAPB1E7.03 | *rpc82*^+^ | HU | SPCPB16A4.04c | *trm8*^+^ | HU |
| SPAPB24D3.04c | *mag1*^+^ | HU, MMS | SPCPB1C11.03 |  | HU |

Deletions were grown in YES medium overnight, and then inoculated into 1 ml YES medium containing different reagents at an *A*_600_ of 0.2. After 24 hours of incubation at 32°C, *A*_600_ were measured and compared to those of no reagent controls. Deletions with *A*_600_ that dropped by 5 fold or more upon reagent treatment were designated as sensitive.

* Genes identified by another global screen in fission yeast. (DNA Repair (Amst). 2009; 8(5):672-679.).

### Table S2 – GO profiling of 52 genes whose deletion mutants showed strong sensitivity to DNA damage reagents (*P*  0.05).

| GO ID | GO Term | Corrected *P*-value | Gene annotation | Total gene annotation |
| --- | --- | --- | --- | --- |
| **1. Cell cycle related process** | | | | |
| GO:0022402 | cell cycle process | 0.009336 | 18 | 592 |
| GO:0022403 | cell cycle phase | 0.011173 | 17 | 546 |
| GO:0000279 | M phase | 0.016957 | 16 | 515 |
| GO:0007049 | cell cycle | 0.023408 | 19 | 719 |
| GO:0051321 | meiotic cell cycle | 0.044061 | 12 | 353 |
| GO:0051327 | M phase of meiotic cell cycle | 0.044184 | 12 | 351 |
| GO:0007126 | Meiosis | 0.044184 | 12 | 351 |
| **2. Chromosome related process** | | | | |
| GO:0006338 | chromatin remodeling | 5.67E-04 | 12 | 157 |
| GO:0051276 | chromosome organization | 0.00155 | 16 | 355 |
| GO:0016568 | chromatin modification | 0.001755 | 12 | 197 |
| GO:0006325 | chromatin organization | 0.003439 | 12 | 218 |
| GO:0016570 | histone modification | 0.008989 | 8 | 102 |
| GO:0016569 | covalent chromatin modification | 0.008989 | 8 | 102 |
| GO:0016571 | histone methylation | 0.043238 | 4 | 21 |
| **3. Macromolecule metabolism and cellular biosynthesis** | | | | |
| GO:0009890 | negative regulation of biosynthetic process | 0.008099 | 10 | 178 |
| GO:0031327 | negative regulation of cellular biosynthetic process | 0.008617 | 10 | 177 |
| GO:0051172 | negative regulation of nitrogen compound metabolic process | 0.010146 | 10 | 175 |
| GO:0045934 | negative regulation of nucleobase, nucleoside, nucleotide and nucleic acid metabolic process | 0.011321 | 10 | 174 |
| GO:0010558 | negative regulation of macromolecule biosynthetic process | 0.011878 | 10 | 171 |
| GO:0010605 | negative regulation of macromolecule metabolic process | 0.012874 | 10 | 196 |
| GO:0006259 | DNA metabolic process | 0.037419 | 12 | 337 |
| **4. Gene expression** | | | | |
| GO:0045892 | negative regulation of transcription, DNA-dependent | 0.048255 | 7 | 119 |
| GO:0016481 | negative regulation of transcription | 0.048256 | 7 | 120 |
| GO:0045814 | negative regulation of gene expression, epigenetic | 0.049375 | 6 | 84 |
| GO:0006342 | chromatin silencing | 0.049375 | 6 | 84 |
| **5. Reproductive process** | | | | |
| GO:0048610 | reproductive cellular process | 0.041024 | 8 | 150 |
| GO:0032505 | reproduction of a single-celled organism | 0.046317 | 7 | 117 |
| GO:0022413 | reproductive process in single-celled organism | 0.046317 | 7 | 117 |
| GO:0003006 | reproductive developmental process | 0.048256 | 7 | 120 |

### Table S3 – Flow cytometry analysis of 37 mutants.

| Mutants | YES (%) | | | YES+HU (%) | | | YES+BLM (%) | | | YES+MMS (%) | | | YES+CPT (%) | | | YES+TBZ (%) | | | YES+UV (%) | | |
| --- | --- | --- | --- | --- | --- | --- | --- | --- | --- | --- | --- | --- | --- | --- | --- | --- | --- | --- | --- | --- | --- |
|  | 1C | 2C | 4C | 1C | 2C | 4C | 1C | 2C | 4C | 1C | 2C | 4C | 1C | 2C | 4C | 1C | 2C | 4C | 1C | 2C | 4C |
| WT | 0.9±0.5 | 97±1 | 3±1 | 0.1±0.03 | 97±1 | 3±1 | 0.3±0.1 | 97±1 | 2±1 | 0.5±0.3 | 98±1 | 1±0.2 | 0.6±0.4 | 97±0.3 | 3±0.2 | 0.2±0.1 | 96±1 | 4±1 | 0.5±0.05 | 97±0.4 | 2±0.4 |
| 1. **Group “2C”** | | | | | | | | | | | | | | | | | | | | | |
| *trk1*Δ | 0.4±0.2 | 96±0.1 | 4±0.3 | 45±3 | 53±3 | 2±0.1 | 1±0.6 | 97±0.4 | 2±0.2 | ND^a^ | | | 1±0.1 | 96±0.1 | 3±0.1 | 1±0.4 | 92±1 | 7±1 | 10±7 | 83±6 | 7±1 |
| *ash2*Δ | 0.5±0.2 | 91±0.4 | 8±1 | 44±4 | 53±4.1 | 2±0.5 | 2±0.4 | 84±1 | 13±0.2 | ND | | | ND | | | 0.7±0.1 | 84±1 | 14±1 | ND | | |
| *scd1*Δ | 3±0.6 | 90±1 | 7±0.1 | 43±5 | 53±4 | 4±1 | 0.9±0.1 | 95±0.2 | 4±0.3 | ND | | | ND | | | ND | | | ND | | |
| *sen1*Δ | 0.3±0.1 | 94±0.2 | 6±0.1 | 22±1 | 76±1.2 | 2±0.5 | 0.7±0.1 | 96±0.6 | 4±0.6 | ND | | | ND | | | 0.5±0.2 | 95±1 | 4±1 | 31±4 | 66±3 | 3±1 |
| *mug61*Δ | 2±0.8 | 89±1 | 8±2 | 58±1 | 39±1 | 3±0.3 | 1±0.2 | 94±0.6 | 5±0.5 | ND | | | ND | | | ND | | | 2±0.4 | 93±1 | 4±0.3 |
| *set1*Δ | 0.4±0.03 | 91±0.5 | 9±0.5 | 13±2 | 85±2 | 2±0.5 | 1±0.6 | 85±0.5 | 14±1 | 7±2 | 79±12 | 14±12 | ND | | | 0.8±0.1 | 84±1 | 15±1 | ND | | |
| *rps2401*Δ | 1±0.1 | 94±3 | 5±3 | 13±2 | 85±2 | 2±0.2 | 3±2 | 93±2 | 4±1 | 12±1 | 85±1 | 3±0.2 | ND | | | ND | | | 9±0.5 | 88±0.1 | 4±0.5 |
| *tcg1*Δ | 0.6±0.5 | 91±1 | 9±2 | 14±2 | 83±2 | 3±0.1 | 3±0.5 | 89±2 | 8±2 | ND | | | ND | | | 0.2±0.1 | 87±1 | 12±1 | ND | | |
| *cch1*Δ | 0.5±0.2 | 92±0.4 | 8±0.2 | 43±3 | 55±3 | 2±0.2 | ND | | | 5±2 | 89±1 | 7±2 | ND | | | ND | | | 35±3 | 62±3 | 4±0.3 |
| *hrp3*Δ | 1±0.1 | 95±1 | 4±1 | ND | | | 4±1 | 95±1 | 1±0.1 | ND | | | ND | | | 3±0.3 | 94±1 | 3±0.3 | 9±1 | 89±2 | 2±0.4 |
| *ers1*Δ | 2±0.3 | 89±0.4 | 9±0.3 | 41±3 | 55±3 | 4±0.4 | ND | | | 12±0.4 | 76±1 | 12±1 | ND | | | 3±0.4 | 85±1 | 12±0.2 | 5±1 | 88±0.4 | 7±1 |
| *rhp55*Δ | 4±0.5 | 90±1 | 6±1 | 2±0.3 | 88±1 | 11±1 | 5±1 | 91±1 | 4±1 | 14±2 | 81±2 | 5±1 | ND | | | 4±0.6 | 89±1 | 7±0.1 | 5±1 | 91±1 | 4±0.6 |
| *myo1*Δ | 1±0.1 | 91±1 | 9±1 | 18±2 | 79±2 | 3±0.3 | 5±0.5 | 87±1 | 8±1 | ND | | | ND | | | 1±0.2 | 69±4 | 31±4 | 1±0.1 | 88±1 | 11±1 |
| *SPAC3F10.17*Δ | 0.1±0.02 | 97±0.3 | 3±0.2 | 36±1 | 63±1 | 1±0.1 | 1±0.3 | 97±1 | 2±0.5 | ND | | | ND | | | ND | | | ND | | |
| *pst2*Δ | 2±0.1 | 94±1 | 4±1 | 26±1 | 72±1 | 2±0.4 | 3±1 | 92±0.3 | 5±1 | 2±0.4 | 92±0.4 | 6±0.4 | ND | | | 3±0.4 | 92±1 | 5±0.3 | 4±0.4 | 92±0.4 | 4±0.1 |
| *gcn5*Δ | 2±0.3 | 96±0.3 | 3±0.1 | 29±3 | 69±3 | 2±0.1 | ND | | | 2±0.1 | 95±1 | 3±1 | 7±1.4 | 86±2 | 7±1 | 2±0.1 | 96±0.3 | 3±0.2 | ND | | |
| 1. **Group “1C”** | | | | | | | | | | | | | | | | | | | | | |
| *clr4*Δ | 18±9 | 75±8 | 7±2 | 34±2 | 58±2 | 8±0.6 | ND | | | 26±2 | 70±3 | 4±0.5 | ND | | | 12±3 | 77±3 | 10±1 | ND | | |
| *snf5*Δ | 10±1 | 87±1 | 4±1 | 23±1 | 74±1 | 3±1 | ND | | | 5±1 | 93±1 | 3±0.3 | ND | | | ND | | | ND | | |
| *rad1*Δ | 6±0.3 | 89±1 | 5±0.3 | 22±9 | 74±9 | 4±0.4 | 2±0.2 | 93±1 | 5±1 | 1±0.2 | 93±1 | 6±1 | 1±0.5 | 93±1 | 6±1 | ND | | | 3±0.2 | 94±1 | 3±1 |
| *rep2*Δ | 12±2 | 81±3 | 7±0.6 | 76±2 | 23±1 | 2±0.2 | 75±1 | 24±1 | 2±0.2 | 72±3 | 26±3 | 2±0.3 | ND | | | ND | | | 74±2 | 24±1 | ±0.2 |
| *srs2*Δ | 9±2 | 88±1 | 2±0.5 | ND | | | 12±2 | 87±2 | 1±0.2 | 3±0.2 | 92±1 | 5±1 | ND | | | ND | | | 3±1 | 95±1 | 3±0.4 |
| *SPBC2A9.02*Δ | 19±1 | 78±1 | 3±0.8 | 18±1 | 82±1 | 1±0.5 | ND | | | ND | | | ND | | | ND | | | 9±1 | 89±1 | 2±0.2 |
| *arz1*Δ | 24±0.1 | 75±0.2 | 1±0.2 | ND | | | ND | | | ND | | | ND | | | ND | | | 25±1 | 74±1 | 1±0.1 |
| *upl2*Δ | 13±2 | 86±2 | 1±0.02 | 1±0.1 | 95±2 | 4±2 | 1±0.5 | 96±1 | 3±0.6 | 4±0.6 | 95±1 | 2±0.3 | ND | | | 2±0.5 | 96±0.3 | 2±0.4 | 4±0.3 | 94±0.2 | 2±0.3 |
| *SPAC27D7.08c*Δ | 18±1 | 79±0.4 | 2±0.8 | 8±2 | 90±2 | 2±0.3 | 5±2 | 92±1 | 3±0.3 | ND | | | ND | | | ND | | | ND | | |
| 1. **Group “W4C”** | | | | | | | | | | | | | | | | | | | | | |
| *mlo3*Δ | 3±0.4 | 86±1 | 11±1 | 7±1 | 87±1 | 6±0.4 | 3±0.2 | 84±0.4 | 13±1 | 8±0.6 | 86±1 | 7±0.3 | ND | | | ND | | | 9±1 | 81±1 | 11±2 |
| *sgf73*Δ | 0.4±0.5 | 71±5 | 28±4 | 9±0.4 | 86±0.1 | 6±1 | ND | | | 7±0.3 | 80±1 | 13±0.4 | ND | | | 0.3±0.01 | 79±1 | 20±1 | ND | | |
| *aah3*Δ | 0.3±0.03 | 83±2 | 17±2 | ND | | | 0.1±0.02 | 87±1 | 13±1 | ND | | | ND | | | 0.2±0.1 | 81±1 | 19±1 | 4±1 | 88±2 | 8±1 |
| *SPCC830.06*Δ | 0.2±0.2 | 65±2 | 34±2 | 28±3 | 52±2 | 20±1 | 0.1±0.1 | 68±1 | 32±1 | ND | | | ND | | | 0.1±0.03 | 72±1 | 27±1 | ND | | |
| *set3*Δ | 0.4±0.03 | 88±2 | 12±2 | ND | | | 0.4±0.2 | 92±1 | 8±1 | 2±0.2 | 92±1 | 6±1 | ND | | | ND | | | 1±0.1 | 90±1 | 8±1 |
| *vac7*Δ | 0.4±0.1 | 73±4 | 26±3 | 1±0.2 | 85±1 | 14±1 | ND | | | 0.2±0.02 | 78±2 | 22±2 | ND | | | 0.1±0.1 | 77±3 | 23±3 | ND | | |
| *atb2*Δ | 1±0.1 | 89±0.2 | 10±0.2 | 4±1 | 93±1 | 3±0.4 | 0.7±0.2 | 92±1 | 7±1 | ND | | | ND | | | 0.6±0.1 | 85±1 | 14±1 | ND | | |
| *meu29*Δ | 0.1±0.01 | 65±3 | 35±3 | 0.1±0.1 | 70±2 | 30±2 | 0.6±0.3 | 70±3 | 30±2 | ND | | | ND | | | 0.1±0.01 | 62±0.3 | 38±0.3 | ND | | |
| 1. **Group “S4C”** | | | | | | | | | | | | | | | | | | | | | |
| *SPBC409.15*Δ | 0 | 8±2 | 92±2 | 0 | 4±0.1 | 96±0.2 | ND | | | 0.7±0.4 | 23±1 | 76±1 | ND | | | 0 | 8±2 | 82±2 | 0.4±0.2 | 13±2 | 87±2 |
| *sec65*Δ | 0.04±0.03 | 7±1 | 93±1 | ND | | | 0.2±0.1 | 9±1 | 91±1 | ND | | | ND | | | 0.02±0.03 | 3±0.4 | 96±0.4 | 0.2±0.03 | 12±1 | 88±2 |
| *spt20*Δ | 0.02±0.01 | 18±1 | 82±1 | 2±1 | 50±2 | 48±2 | ND | | | ND | | | 0 | 20±3 | 80±3 | ND | | | ND | | |
| *pab1*Δ | 0 | 7±1 | 92±0.4 | 0.1±0.1 | 23±3 | 77±3 | ND | | | 0.01±0.01 | 21±3 | 79±3 | ND | | | 0.01±0.01 | 9±1 | 91±1 | 0.5±0.03 | 11±1 | 89±1 |

^a^ ND Not detected.

Cells were grown to the logarithmic phase and treated with DNA damage reagent for 2 hr. For UV sensitivity assay, cells were exposed to 60 J/m^2^ radiation and then grown for 2 h. Cells were harvested afterward and subjected to cytometry analysis. Fractions of 1C, 2C and 4C DNA content were calculated by Flowjo 2.0. Data were presented as average from three independent experiments.

### Table S4 – Primers used for real time PCR analysis in this study.

| Primer | Sequence (5’→3’) |
| --- | --- |
| *ACT1*+ | TTGATAATGGCTCTGGTATGTGC |
| *ACT1*− | CGACCAGAGGCATACAAAGACA |
| *ABP1*+ | TCGGGCTGCTAATGTAAAACC |
| *ABP1*− | GGCTGGGAAATAGAGGCTGA |
| *ABP2*+ | GTTTGAACCACCTGACGAAGAC |
| *ABP2*− | GCAGAGCGAAGCATTGTATTTT |
| *ACE2*+ | CCTCGGGTCCTGCCTCTT |
| *ACE2*− | TATGCCTGCATACATCCAAATG |
| *AGN1*+ | TCGGTTATTCTGATGTTAGCACG |
| *AGN1*− | CCAAGGCGAGACTGGTGC |
| *ENG1*+ | TCTTCTTCTACCACCACGACCTC |
| *ENG1*− | CAGCCATATTGCGAAGGGTT |
| *CDC18*+ | CAATCTCTGAAGTAAGCGATGATAGTAT |
| *CDC18*− | ACAACAAGCGTACAAAGGATGG |
| *CDT1*+ | CGCGTAAGCTAACATCATCTCA |
| *CDT1*− | CAATCCTGCTGAATGTGACTACTT |

### Figure S1 – Spot assay of 52 deletions.

Exponentially growing cells, WT or deletions, were harvested and 5-fold serial dilutions were spotted on the plates supplemented with DNA damage reagents. The plates were photographed after 3~4 days of incubation at 32°C.


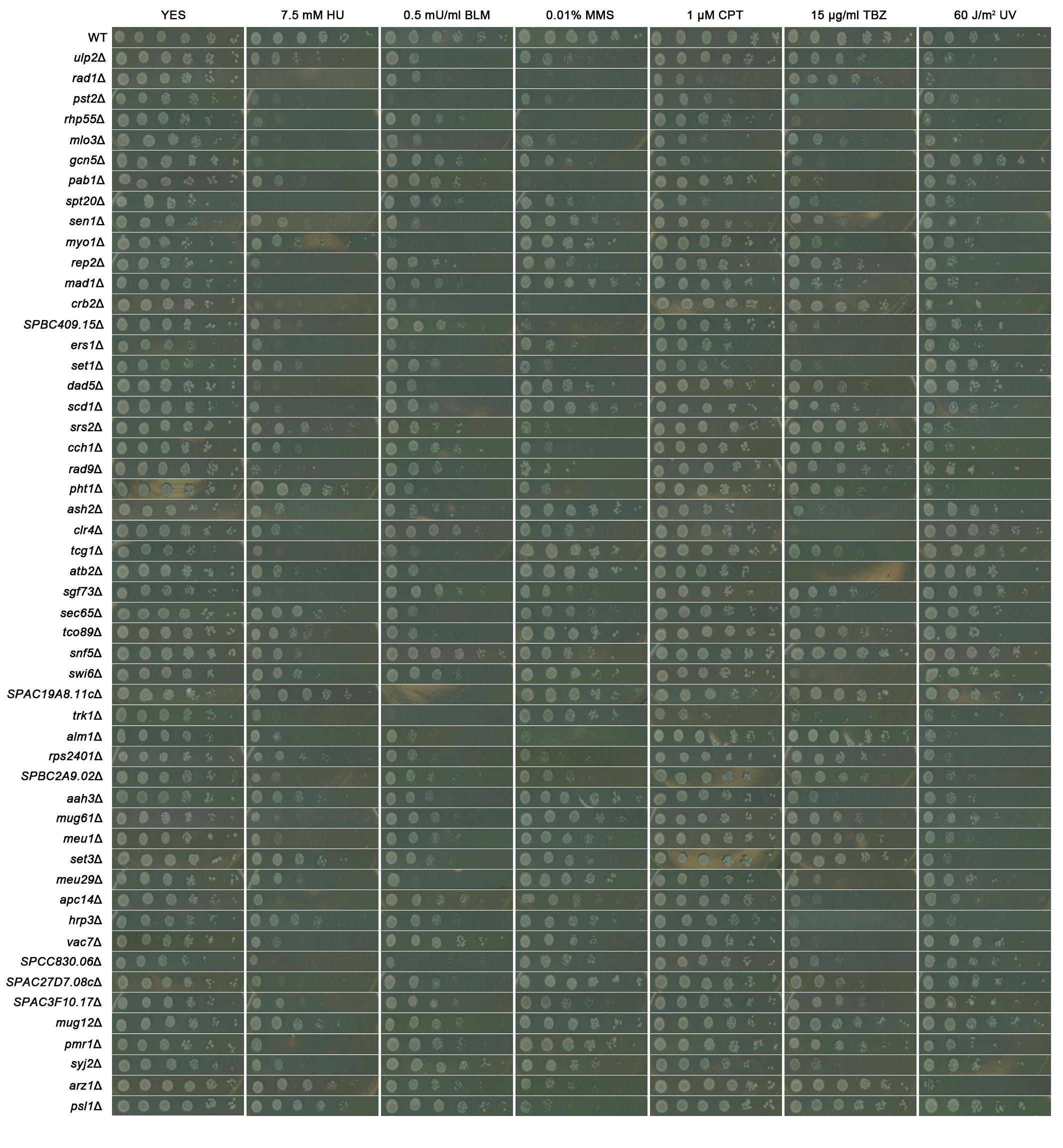


### Figure S2 – Flow cytometry analysis of deletions in “2C” group.


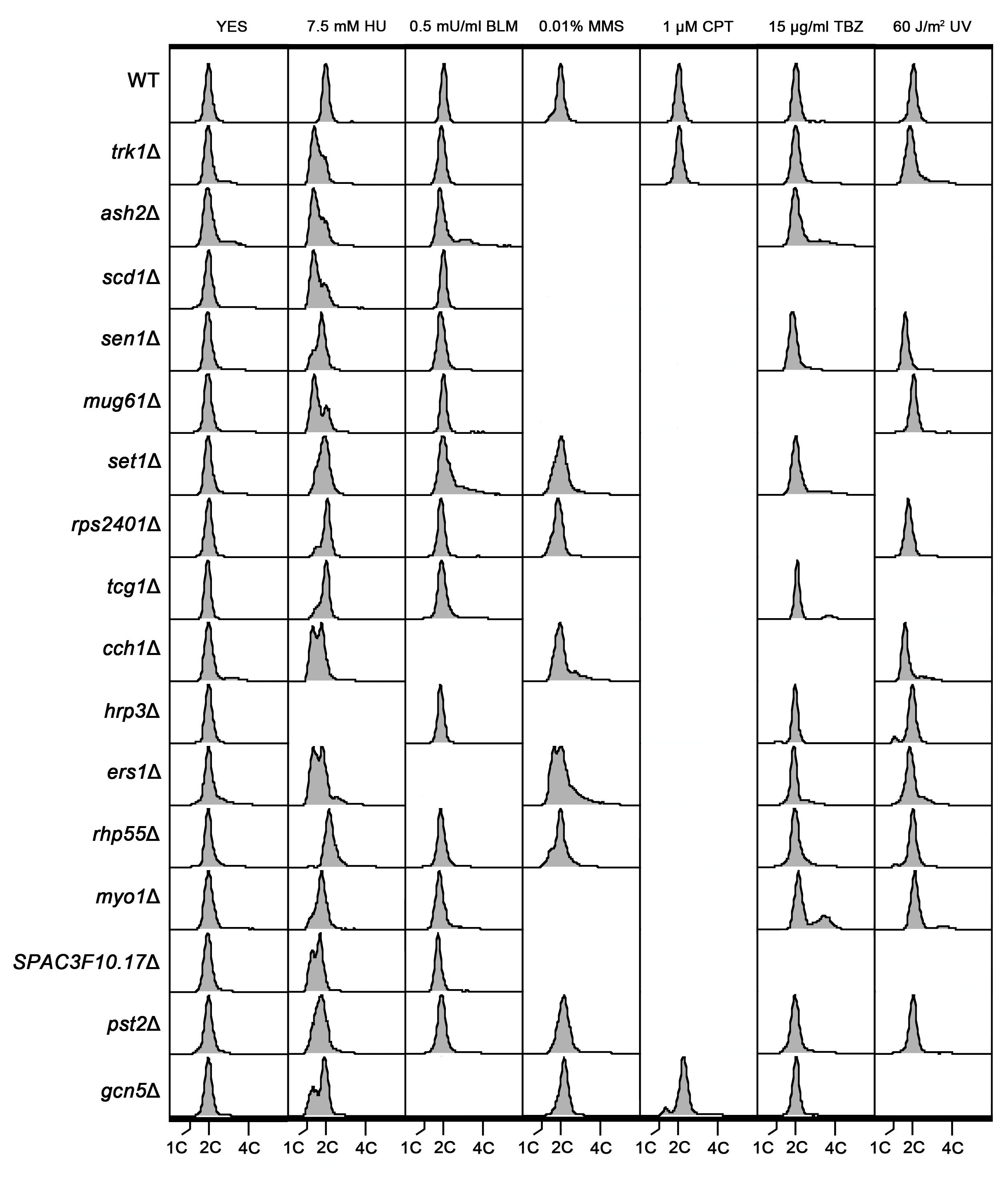


### Figure S3 – Flow cytometry analysis of deletions in “1C” group.


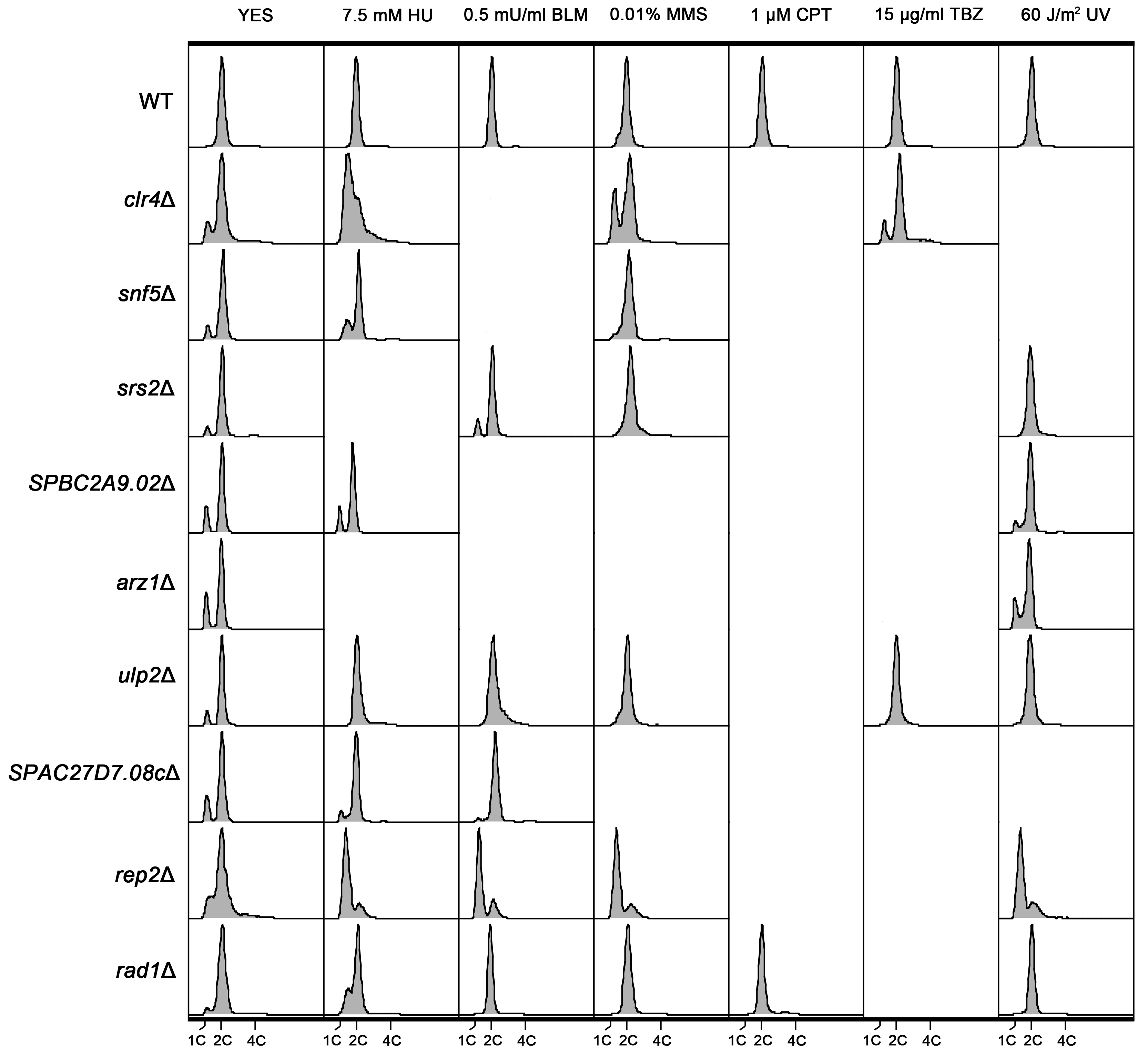


### Figure S4 – Flow cytometry analysis of deletions in “W4C” group.


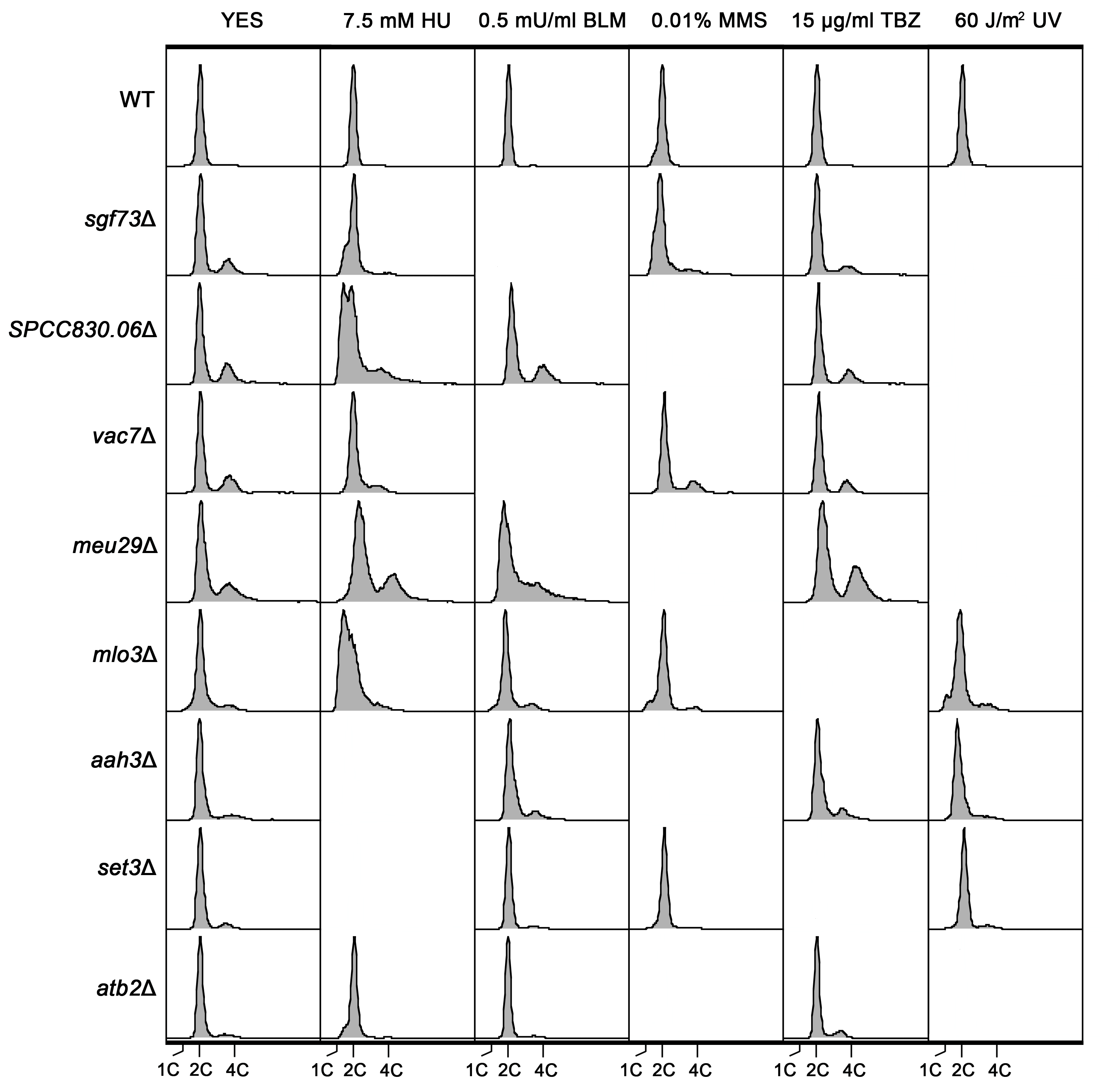


### Figure S5 – Flow cytometry analysis of deletions in “S4C” group.


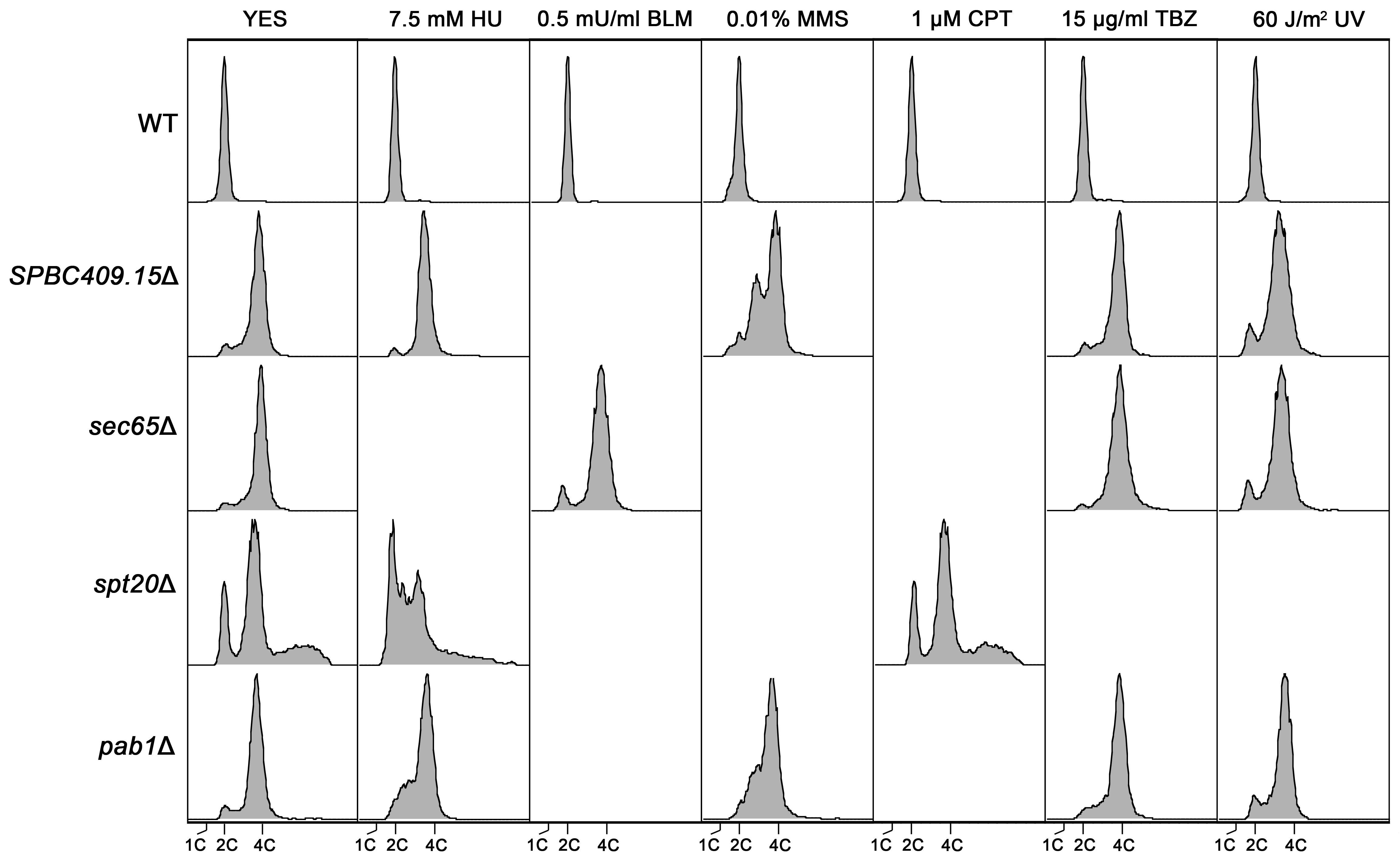

Supplement: Additional file 1 — Table S1. List of genes whose deletions exhibited sensitivity to DNA damage reagents during the second round of screen. Table S2. GO profiling of 52 genes whose deletion mutants showed strong sensitivity to DNA damage reagents (P ≤ 0.05). Table S3. Flow cytometry analysis of 37 mutants. Table S4. Primers used for real time PCR analysis in this study. Figure S1. Spot assay of 52 deletions. Exponentially growing cells, WT or deletions, were harvested and 5-fold serial dilutions were spotted on the plates supplemented with DNA damage reagents. The plates were photographed after 3~4 days of incubation at 32°C. Figure S2. Flow cytometry analysis of deletions in “2C” group. Figure S3. Flow cytometry analysis of deletions in “1C” group. Figure S4. Flow cytometry analysis of deletions in “W4C” group. Figure S5. Flow cytometry analysis of deletions in “S4C” group. [file 1471-2164-13-662-S1.doc]
